# Incidence of Hospitalization for Heart Failure and Case-Fatality Among 3.25 Million People With and Without Diabetes Mellitus

**DOI:** 10.1161/CIRCULATIONAHA.118.034986

**Published:** 2018-12-10

**Authors:** David A. McAllister, Stephanie H. Read, Jan Kerssens, Shona Livingstone, Stuart McGurnaghan, Pardeep Jhund, John Petrie, Naveed Sattar, Colin Fischbacher, Soren Lund Kristensen, John McMurray, Helen M. Colhoun, Sarah H. Wild

**Affiliations:** 1 Institute of Health and Wellbeing (D.A.M.), University of Glasgow, United Kingdom.; 2 Institute of Cardiovascular and Medical Sciences (P.J., J.P., N.S., J.M.), University of Glasgow, United Kingdom.; 3 NHS National Services Scotland, Edinburgh, United Kingdom (D.A.M., J.K., C.F.).; 4 Usher Institute of Population Health Sciences and Informatics (S.H.R, S.H.W).; 5 MRC Institute of Genetics and Molecular Medicine (S.M., H.M.C).; 6 School of Medicine, University of Dundee, United Kingdom (S.L.).; 7 Bispebjerg Hospital, Copenhagen University, Denmark (S.L.K.).

**Keywords:** diabetes mellitus, electronic health records, epidemiology, heart failure, incidence, mortality, registries

## Abstract

**Background:**

Recent clinical trials of new glucose-lowering treatments have drawn attention to the importance of hospitalization for heart failure as a complication of diabetes mellitus. However, the epidemiology is not well described, particularly for type 1 diabetes mellitus. We examined the incidence and case-fatality of heart failure hospitalizations in the entire population aged ≥30 years resident in Scotland during 2004 to 2013.

**Methods:**

Date and type of diabetes mellitus diagnosis were linked to heart failure hospitalizations and deaths using the national Scottish registers. Incidence rates and case-fatality were estimated in regression models (quasi-Poisson and logistic regression respectively). All estimates are adjusted for age, sex, socioeconomic status, and calendar-year.

**Results:**

Over the 10-year period of the study, among 3.25 million people there were 91, 429, 22 959, and 1313 incident heart failure events among those without diabetes mellitus, with type 2, and type 1 diabetes mellitus, respectively. The crude incidence rates of heart failure hospitalization were therefore 2.4, 12.4, and 5.6 per 1000 person-years for these 3 groups. Heart failure hospitalization incidence was higher in people with diabetes mellitus, regardless of type, than in people without. Relative differences were smallest for older men, in whom the difference was nonetheless large (men aged 80, rate ratio 1.78; 95% CI, 1.45–2.19). Rates declined similarly, by 0.2% per calendar-year, in people with type 2 diabetes mellitus and without diabetes mellitus. Rates fell faster, however, in those with type 1 diabetes mellitus (2.2% per calendar-year, rate ratio for type 1/calendar-year interaction 0.978; 95% CI, 0.959–0.998).

Thirty-day case-fatality was similar among people with type 2 diabetes mellitus and without diabetes mellitus, but was higher in type 1 diabetes mellitus for men (odds ratio, 0.96; 95% CI, 0.95–0.96) and women (odds ratio, 0.98; 95% CI, 0.97–0.98). Case-fatality declined over time for all groups (3.3% per calendar-year, odds ratio per calendar-year 0.967; 95% CI, 0.961–0.973).

**Conclusions:**

Despite falling incidence, particularly in type 1 diabetes mellitus, heart failure remains ≈2-fold higher than in people without diabetes mellitus, with higher case-fatality in those with type 1 diabetes mellitus. These findings support the view that heart failure is an under-recognized and important complication in diabetes mellitus, particularly for type 1 disease.

## Abstract 2

Supplemental Digital Content is available in the text.

**Clinical Perspective**

**What Is New?**

- Heart failure incidence has fallen over time for people with and without diabetes mellitus, but is ≈2 times higher in people with diabetes mellitus than people without diabetes mellitus.
- Heart failure case-fatality is higher in people with type 1 diabetes mellitus.
- Duration of diabetes mellitus and glycated hemoglobin was associated with increased risk of heart failure in type 1 and type 2 diabetes mellitus.

**What Are the Clinical Implications?**

- Clinicians should be aware of the importance of heart failure in diabetes mellitus, especially in type 1 diabetes mellitus, where this risk is underappreciated.

Recent clinical trials of new glucose-lowering treatments have drawn attention to the importance of hospitalization for heart failure as a complication of diabetes mellitus.^1–4^ However, little is known about the epidemiology of heart failure in unselected individuals with type 2 diabetes mellitus, and even less about those with type 1 diabetes mellitus.

We have examined the incidence of heart failure for an entire country, and how this has changed over time. All residents in Scotland receive care from the National Health Service (NHS) which is free at the point of contact. All diabetes mellitus–related community (primary care) and hospital outpatient encounters in Scotland are recorded in a centrally-accessible database, and these are linked to national hospitalization and mortality records. Given the prognostic import of developing heart failure, we have also investigated the case-fatality related to incident heart failure hospitalization and how this has changed over time.

Specifically, we examined the incidence rate for heart failure hospitalization and 30-day case-fatality after heart failure hospitalization over a 10-year period from January 2004 to December 2013 among people with diabetes mellitus (type 1 and type 2).

## Methods

### Access to Data and Methods

The data, analytic methods, and study materials will not be made available to other researchers for purposes of reproducing the results or replicating the procedure. However, individual-level data are available via application to NHS Information Services Scotland,^5^ aggregate data are provided in the supplemental appendix (Tables I and II in the online-only Data Supplement), and analysis code has been posted online.^6^

### Datasets

We used data from the population-based Scottish diabetes mellitus register linked to national hospitalization and death records. The Scottish diabetes mellitus register is derived from primary and secondary care records for people with diabetes mellitus diagnosed in normal clinical practice using blood glucose measurements or hemoglobin A1c with coverage of >99% since 2004,^7^ the start of the study period.

Anyone alive in Scotland aged 30 and older at any time from 2004 to 2013 was included in the analyses.

### Definition of Heart Failure

Heart failure was identified from the first mention in any position (primary or secondary diagnoses) in hospital inpatient records using codes from the ninth (ICD-9) and tenth (ICD-10) International Classification of Diseases revisions (402, 402, 402.1, 402.2, 402.4, 402.6, 402.9, 425.5, 428, 428, 428.1, 428.9 and I11.0, I13.0, I13.2, I42.6, I50.0, I50.1, I50.9, respectively). Outpatient attendances with heart failure are not recorded in the Scottish national healthcare database, nor in the Scottish diabetes mellitus register.

### Diabetes Mellitus Status and Diagnosis Date

Date and type of diabetes mellitus were obtained from the diabetes mellitus register based on the clinician-recorded date of diagnosis. Diabetes mellitus status was defined as type 2 diabetes mellitus, type 1 diabetes mellitus and no diabetes mellitus.

### Covariates

Age at first heart failure hospitalization and sex were identified from hospital admission and mortality records. Socioeconomic status was assessed via an area-based measure of deprivation which is assigned to residents of Scotland on the basis of where they live using the Scottish Index of Multiple Deprivation (SIMD).^8^ SIMD 2009 combines 31 indicators across 7 domains: income, employment, health, education, housing, geographic access, and crime. The index is generated from a weighted sum of the 7 domain scores for each area defined by postcodes (zip codes) which contain a median of 769 people. SIMD data were missing for approximately 1% of hospital admissions or death records, and these records were excluded from the analysis.

### Clinical Risk Factors

As an additional analysis, to explore the association between demographic and clinical risk factors and subsequent risk of heart failure admissions, we also identified a closed cohort from the Scottish Diabetes Register. All patients in this cohort had been diagnosed with type 1 or type 2 diabetes mellitus on or before October 1, 2013 and had not had a heart failure admission in the 10 years before this date. Follow-up data were available for 3 years.

Heart failure in the closed cohort was defined as per the main analysis. However, the clinician-recorded diabetes mellitus type was corrected through the use of additional clinical information (eg, medication use) as described in a previous publication.^9^

For this cohort, baseline characteristics such as glycated hemoglobin, body mass index, duration of diabetes mellitus, blood pressure, and retinopathy were also obtained from the diabetes mellitus register, taking the mean (or mode) of all measures not more than 3 years before the start date. Data on current prescriptions for selected drug classes (Table III in the online-only Data Supplement) were also obtained, as were data on previous stroke and myocardial infarction within the previous 10 years.

### Statistical Analysis

#### Hospitalizations and Deaths

Using the population-based Scottish diabetes mellitus register, linked to national hospitalization and death records, heart failure hospitalizations from January 1, 2004 (or the date at which each person was diagnosed with diabetes mellitus, if this occurred during the study period) to December 31, 2013 were identified for people with diabetes mellitus aged 20 to 89 years. Heart failure hospitalizations were defined as incident if these were the first to occur on or after January 2004 where no previous heart failure hospitalization had been recorded during the preceding 10 years (Figure I in the online-only Data Supplement). As with our previous analyses we used a fixed period to define incident cases to avoid bias from nonobservation of prevalent cases.^10^ This analysis was replicated using the entire national hospitalization and death record to identify heart failure hospitalizations for the general adult population. Incident hospitalizations were summed by calendar year, age, sex, deprivation decile, and, for the diabetes mellitus dataset, diabetes mellitus type. Within the levels of these stratifying variables, the number of incident heart failure hospitalizations in people without diabetes mellitus was derived by subtracting the diabetes mellitus heart failure hospitalizations from the general population heart failure hospitalizations.

Case-fatality was examined at 30 days, defined as the proportion of patients who died from any cause within 30 days of any heart failure hospitalization. Both in-hospital and out of hospital deaths were included.

#### Person-Time

As for hospitalizations, person-time for people with diabetes mellitus was estimated using the linked diabetes mellitus register, hospitalization, and death data. Each individual’s person-time was estimated as the number of days from the start of the study period (or date at which each person was diagnosed with diabetes mellitus, if this occurred during the study period) to the date of the incident heart failure hospitalization, death, or censoring at December 31, 2013 (Tables IV and V in the online-only Data Supplement). This was summed by diabetes mellitus type, calendar-year, age, sex, and deprivation decile. Mid-year population estimates for the general population in Scotland, stratified by age, sex, and deprivation, were obtained from National Records of Scotland. For people without diabetes mellitus, person-time not at risk (attributable to prevalent or incident heart failure events) was also summed by the same stratifying variables. Within the levels of these stratifying variables, person-time for people with no diabetes mellitus was obtained by subtracting the diabetes mellitus person-time from the midyear estimates population estimates.

#### Modeling

Heart failure incidence rates were estimated by age, sex, deprivation, calendar year, and diabetes mellitus status. Confidence intervals for the rate ratios and rate differences were obtained as per Rothman et al.^11^

For the main analysis, regression models were fit using nonparametric smooth terms (penalized thin plate regression splines) and interaction terms to allow for nonlinearity and heterogeneity respectively. We used regression in preference to stratification to avoid categorizing continuous variables, which can result in unstable estimates if a stratum is too small, and loss of information if a stratum is too heterogeneous.

Interactions are reported on the same scale as the main effects as the rate ratio (RR) or odds ratio (OR), because this can be interpreted as the relative difference in the magnitude of an association per one-unit change in the interacting variable.

For incidence rates, generalized linear models were used with a log-link and Poisson error distribution, using a scaling factor (quasi-Poisson) to allow for overdispersion. Age in years was divided by 10 so that each increment was a decade (eg, age 44 was transformed to 4.4). Deprivation deciles were treated as a deprivation score ranging from 1 to 10, which was divided by 5 such that each increment was a 5-point increment. Terms were included for main effects in all models regardless of significance or magnitude of effect. The large and heterogeneous study population with a large number of events allowed us to investigate whether the magnitude of risk associated with diabetes mellitus varied by biologically plausible and clinically relevant variables on the basis of prior knowledge of diabetes mellitus and heart failure. Interaction terms (between 2 or more of age, sex, deprivation, and diabetes mellitus status) were retained if the exponentiated coefficient (on the transformed scale) was ≥1.05 or ≤0.95. However, for interactions with calendar year, all statistically significant interactions (at *P*<0.05) were included in the final models. Using the same covariates, generalized linear models with a logit-link and binomial error distribution were used to model both heart failure prevalence and case-fatality.

In additional analyses these models were repeated after excluding patients with a previous admission for ischemic heart disease (ICD-9 410–414 or ICD-10 I20–25), and in separate sensitivity analyses after adding additional ICD-10 codes (I42.0, I42.7, I42.8, I42.9) for cardiomyopathy, and after restricting the analysis to admissions where heart failure was the primary diagnosis (recorded in the first of 6 possible diagnostic positions).

For the analysis of the association between the 3-year risk (odds) of heart failure and clinical and demographic characteristics in the closed cohort, we used logistic regression models. Covariate missingness was addressed using multiple imputation (Appendix in the online-only Data Supplement). We fitted models for type 1 and type 2 diabetes mellitus, and a third model which included covariate/diabetes mellitus–type interaction terms to compare risk factor associations between type 1 and type 2 diabetes mellitus. Multiple imputation was used for missing data (Table IV in the online-only Data Supplement).

The association between heart failure and drug prescription was not modeled because of the high likelihood of confounding by indication. However, we did compare the odds of cardiovascular medication prescription in people with type 2 versus type 1 diabetes mellitus. Each drug class was modeled unadjusted, adjusting for age and sex, and for the risk of heart failure (on the logit scale) according to each person’s baseline characteristics. The latter was derived from the model of 3-year risk in the closed cohort described above.

SPSS was used to extract the administrative data, and R version 3.4.3 (Vienna, Austria) was used for the statistical analyses.

Approval for the creation and analysis of the linked dataset containing no person-identifying information was obtained from the Scottish Care Information – Diabetes Collaboration (SCI-DC) steering committee, the Scottish multi-center research ethics committee (reference number 11/AL/0225), the Privacy Advisory Committee of NHS National Services Scotland, and Caldicott guardians.

## Results

This study included the entire Scottish population who were aged 30 or older at any time between 2004 to 2013. In 2004, this comprised 3 066 253 people without a diagnosis of diabetes mellitus, 136 042 with a diagnosis of type 2 diabetes mellitus, and 18 240 with a diagnosis of type 1 diabetes mellitus. Of these, 1 642 022 (53.6%), 63 086 (46.4%), and 8141 (44.6%), respectively, were women and the mean (and SD) ages were 52.9 (15.3), 65.0 (12.2), and 50.0 (14.1) years, respectively. SIMD was similar across the groups with mean (SD) deprivation scores of 5.5 (2.9), 5.9 (2.8), and 5.6 (2.8), respectively.

### Incidence of Heart Failure Hospitalization

People without diabetes mellitus (n=28 681), with type 2 diabetes mellitus (n=3480), and with type 1 diabetes mellitus (n=382) were excluded because of a previous heart failure hospitalization (Figures II and III in the online-only Data Supplement, Table VII in the online-only Data Supplement). Over the 10-year period of the study, among the 3.25 million people remaining there were 91 429, 22 959, and 1313 incident heart failure events (during 38 112 739.9, 1 855 281.8, and 235 924.2 person-years) among those without diabetes mellitus, with type 2 diabetes mellitus, and with type 1 diabetes mellitus, respectively.

**Figure 1.**
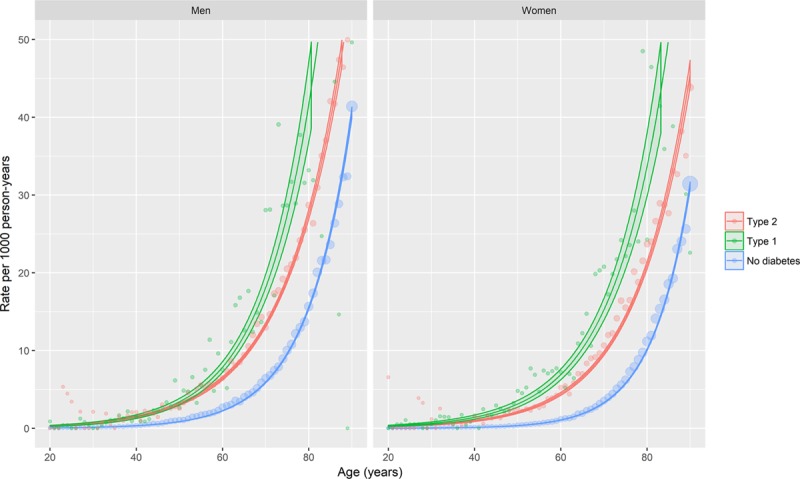
**Age-, sex-, and deprivation-adjusted incidence of heart failure hospitalization by diabetes mellitus type, age, and sex.** The lines represent the predicted rates obtained from quasi-Poisson regression models of incident heart failure events. The ribbons are 95% confidence intervals. Covariates included in the model were age, sex, deprivation, and diabetes mellitus type, with interaction terms included where these improved model fit. Predictions were made at the median deprivation score. Points represent event rates stratified by age (in years), sex, and diabetes mellitus type. Models are given in full in the Appendix in the online-only Data Supplement.

Heart failure incident hospitalization estimated rates from Poisson regression models adjusting for age, sex, and deprivation are shown in Figure 1 (and additionally in Figures IV and V in the online-only Data Supplement and in Table VIII in the online-only Data Supplement). For illustration, age-sex stratified rates for 2013 are shown in Table 1. Rates varied markedly with age and differed according to sex and whether or not diabetes mellitus was present.

**Table 1.**
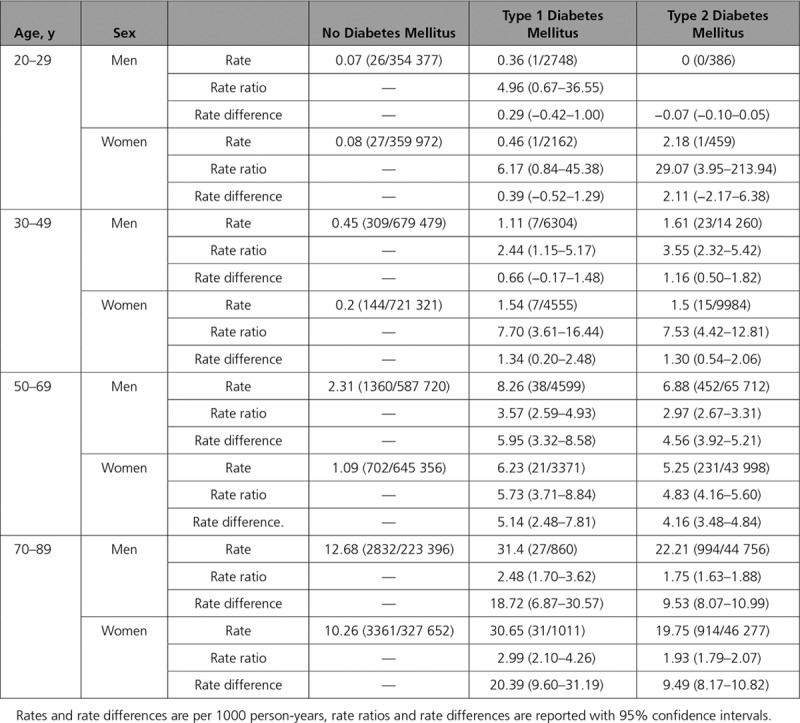
Incidence of Heart Failure Admissions, by Age and Sex

Overall, the rate of incident heart failure hospitalization rose steeply with age, was somewhat higher in men than in women, and was higher in individuals with diabetes mellitus than in those without this diagnosis. Incident heart failure hospitalization was also higher in people with type 1 compared with type 2 diabetes mellitus.

We found the relative risk of diabetes mellitus–related heart failure hospitalization (as indicated by the rate ratio) was highest in younger people and higher in women than men (Figure 1 and Table 1). However, as the absolute rate of incident heart failure was highest in older individuals, the greatest difference in absolute rates were also seen in these groups, and not in younger people and women (Figure 1).

### Time Trends in Incident Heart Failure Hospitalization

Across the period studied, the rate of incident heart failure hospitalization fell slightly, at around 0.2% per calendar-year (RR per calendar-year 0.998; 95% CI, 0.991–1.005).

In models adjusting for age, sex, and deprivation, the rate of decline was slightly steeper in older individuals. The rate of decline was 0.5% per calendar-year faster per 10-year increment in age (RR for interaction 0.995; 95% CI, 0.992–0.998), and was similar in men and women (RR for interaction 1.006; 95% CI, 0.998–1.014), and according to deprivation (RR for interaction [per 5-point increment in deprivation score] 0.996; 95% CI, 0.989–1.003)

**Figure 2.**
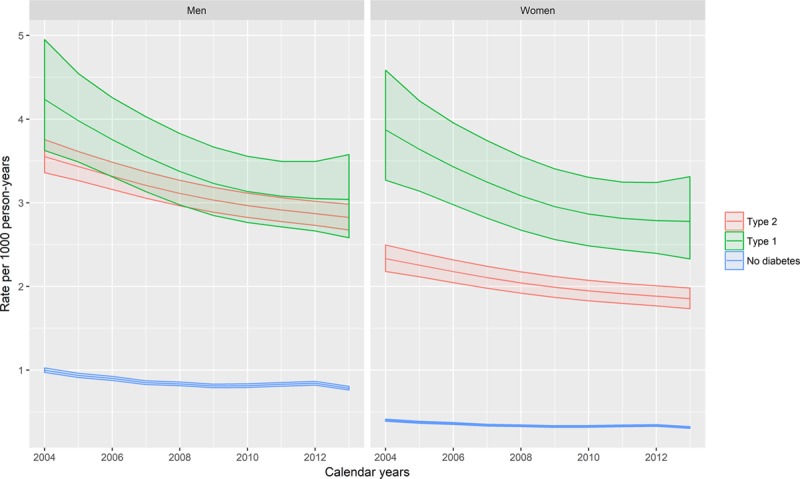
**Age-, sex-, and deprivation-adjusted trends in incident heart failure hospitalization by diabetes mellitus type, sex, and calendar-year.** The lines represent the predicted rates obtained from generalized additive models of incident heart failure events. The ribbons are 95% confidence intervals. Covariates included in the model were age, sex, deprivation, diabetes mellitus type, and calendar year, with interaction terms included where these improved model fit. The model was fit with a log-link and Poisson likelihood, with correction of the standard errors for overdispersion. Penalized thin plate regression splines were used to model nonlinear associations for calendar year by diagnosis type. Predictions were made for men and women aged 50 (as this was the closest decade to the mean age in the general population). Models are given in full in the Appendix in the online-only Data Supplement. See https://ihwph-hehta.shinyapps.io/dm_hf_fig2/ for an interactive version of this plot.

From the same model, trends in heart failure incidence rates in people with type 2 diabetes mellitus were similar to those in individuals without diabetes mellitus over a ten-year period from 2004 (Figure 2, Tables IX and X in the online-only Data Supplement). However, there was some evidence of a more rapid decline in people with type 1 diabetes mellitus, where the decrease was 2.2% per calendar-year faster than in people without diabetes mellitus.

An interactive version of Figure 2, where diabetes mellitus type and sex-specific temporal trends can be shown for patients of any age or with any level of socio-economic deprivation, is available at https://ihwph-hehta.shinyapps.io/dm_hf_fig2/.

Notwithstanding any temporal decrease in absolute rates, the rate ratios for heart failure hospitalization remained large throughout the study period for both types of diabetes mellitus. For example, in 2013 the rate ratios in people with type 2 diabetes mellitus, compared with those with no diabetes mellitus, were 5.81 (95% CI, 4.91–6.86) and 3.55 (95% CI, 2.99–4.21) for 50-year-old women and men, respectively.

### Case-Fatality of Incident Heart Failure Hospitalization

Over the period of the study, 14.2% (16406/115701) of people admitted to hospital with heart failure died within 30 days of admission.

**Figure 3.**
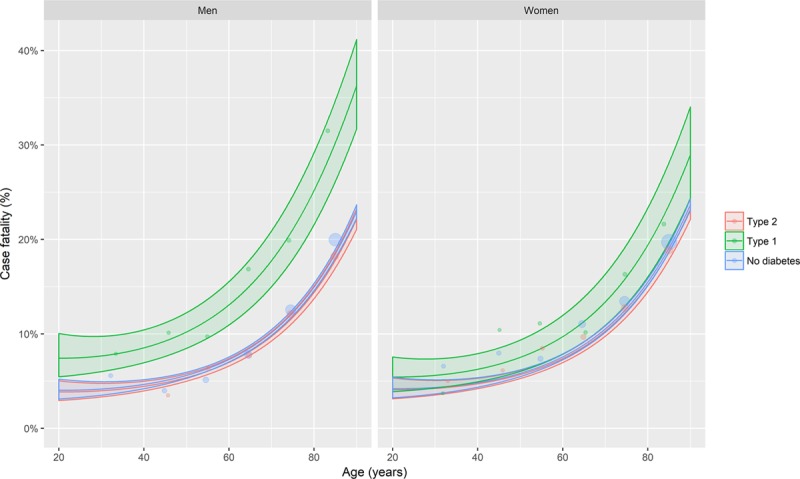
**Age-, sex-, and deprivation-adjusted 30-day case-fatality of incident heart failure hospitalization by age, sex, and diabetes mellitus type.** The lines represent the predicted case-fatality proportions obtained from logistic regression models of death. Covariates included in the model were age, sex, deprivation, and diabetes mellitus type, with interaction terms included where these improved model fit. Predictions were made at the median deprivation score. Points represent case-fatality proportions stratified by age, sex, and diabetes mellitus type, with the point size being proportional to the number in the denominator. Models are given in full in the Appendix in the online-only Data Supplement.

Thirty-day case-fatality results obtained from logistic regression models adjusting for age, sex, and deprivation are shown in Figure 3 (see the Appendix in the online-only Data Supplement for model coefficients, including interactions). For illustration, age–sex stratified case-fatality is shown in Table 2. Case-fatality varied markedly with age and differed according to sex and whether or not type 1 diabetes mellitus was present. It was higher in women than in men and in older people than in younger people. However, it was similar in people with type 2 diabetes mellitus and in people without diabetes mellitus, for men the OR was 0.96 (95% CI, 0.95–0.96), and for women the OR was 0.98 (95% CI, 0.97–0.98).

**Table 2.**
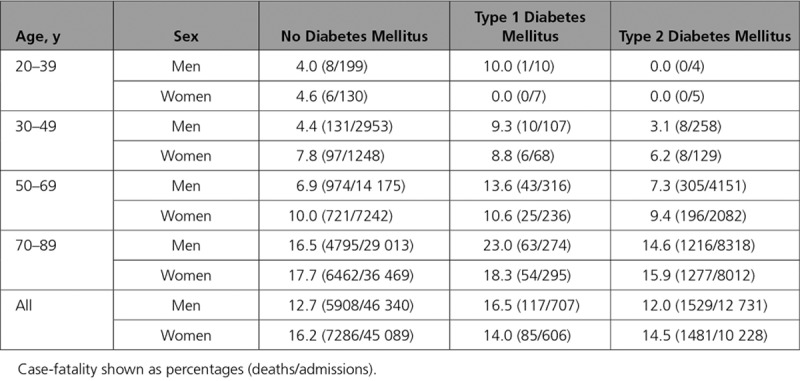
Thirty-Day Case-Fatality of Incident Heart Failure Hospitalization, by Age and Sex

However, case-fatality was higher among people with type 1 diabetes mellitus compared with people without diabetes mellitus; the difference was larger for men (OR, 1.91; 95% CI, 1.68–2.18) than for women (OR, 1.31; 95% CI, 1.05–1.65).

**Figure 4.**
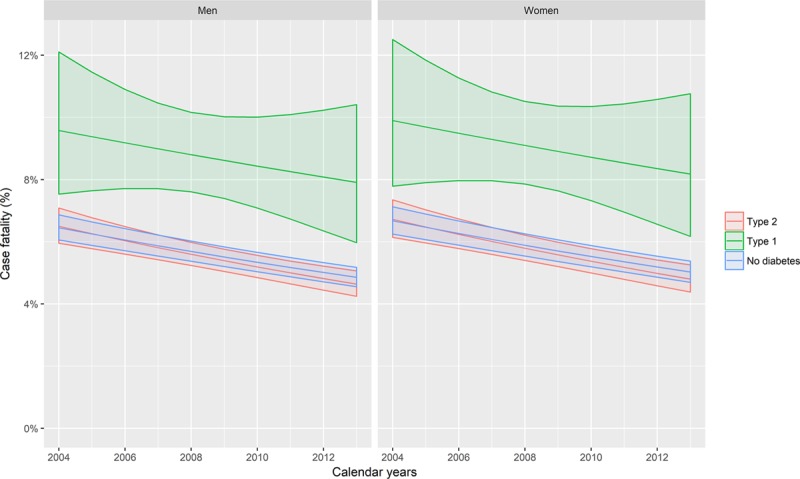
**Age-, sex-, and deprivation-adjusted trends in 30-day case-fatality of incident heart failure hospitalization.** The lines represent the predicted rates obtained from generalized additive models of heart failure 30-day case-fatality on age, sex, deprivation, diabetes mellitus type, and calendar year, with interaction terms included where these improved model fit, using a logit-link and binomial likelihood. Predictions were made for men and women aged 50 (as this was the closest decade to the mean age in the general population). Models are given in full in the Appendix in the online-only Data Supplement.

Trends in case-fatality were also modeled adjusting for age, sex, deprivation, calendar year, and type of diabetes mellitus (Figure 4, Tables XI and XII in the online-only Data Supplement). The rate of decline was around 3.3% per calendar-year (OR per calendar year, 0.967; 95% CI, 0.961–0.974) in people without diabetes mellitus. There was no evidence of a steeper decline in people with type 1 diabetes mellitus (OR for calendar year/type 2 interaction, 1.011; 95% CI, 0.959–1.065) or type 2 diabetes mellitus (OR for calendar year/type 2 interaction, 0.994; 95% CI, 0.979–1.009).

### Incidence of Heart Failure Hospitalization Without Previous Ischemic Heart Disease

**Figure 5.**
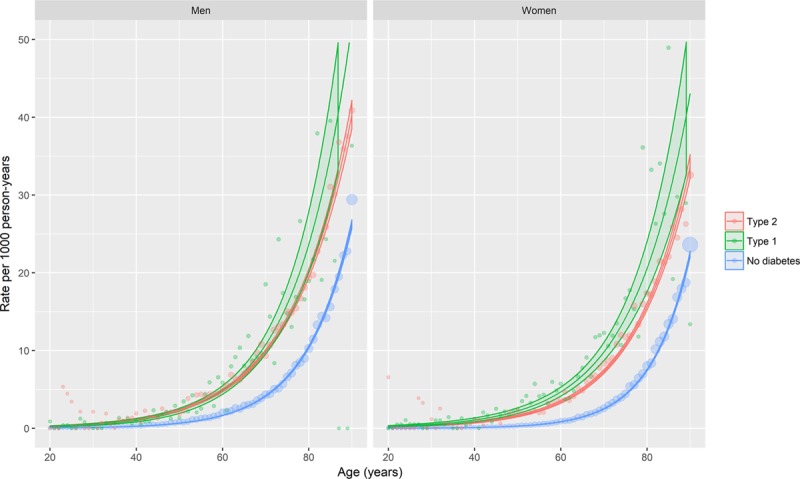
**Age-, sex-, and deprivation-adjusted incidence of heart failure hospitalization by diabetes mellitus type, age, and sex in people without previous ischemic heart disease.** The lines represent the predicted rates obtained from quasi-Poisson regression models of incident heart failure events. The ribbons are 95% confidence intervals. Covariates included in the model were age, sex, deprivation, and diabetes mellitus type, with interaction terms included where these improved model fit. Predictions were made at the median deprivation score. Points represent event rates stratified by age (in years), sex, and diabetes mellitus type. Models are given in full in the Appendix in the online-only Data Supplement.

Incident heart failure hospitalization was lower in people who had never had a previous admission for ischemic heart disease admission (ICD-9 410–414 or ICD-10 I20-25), with rates per 1000 person-years (py) of 1.7 (65 657 events/38 117 663.1 py), 9.2 (17 175 events/1 867 390.1 py), and 3.6 (841 events/236 843.7 py) for the no-diabetes mellitus, type 2 diabetes mellitus, and type 1 diabetes mellitus groups, respectively. Nevertheless, the rate ratios for both type 1 and type 2 diabetes mellitus in this group (Figures 5, Figures VI and VII in the online-only Data Supplement, and Tables XIII–XVI in the online-only Data Supplement) were similar to those in the whole cohort (Figure 1).

### Risk Factors for Heart Failure in People With Diabetes Mellitus

Two hundred thousand, two hundred eight people with type 2 diabetes mellitus and 26,189 with type 1 had not had a previous hospital admission with heart failure on October 1, 2013. In this cohort, over 3 years of follow-up the risk of heart failure was 6752 (2.3%) and 231 (0.9%) for type 2 and type 1 diabetes mellitus, respectively.

Age, sex, body mass index, estimated glomerular filtration rate, smoking, previous ischemic heart disease, and stroke all predicted an increased risk of heart failure, although no associations were found for systolic blood pressure, total cholesterol, or HDL cholesterol (Table 3). After adjusting for conventional risk factors, longer duration of diabetes mellitus and higher concentrations of hemoglobin A1c also predicted heart failure risk. Of note, there was no evidence that any of the predictors of heart failure differed between type 1 and type 2 diabetes mellitus.

**Table 3.**
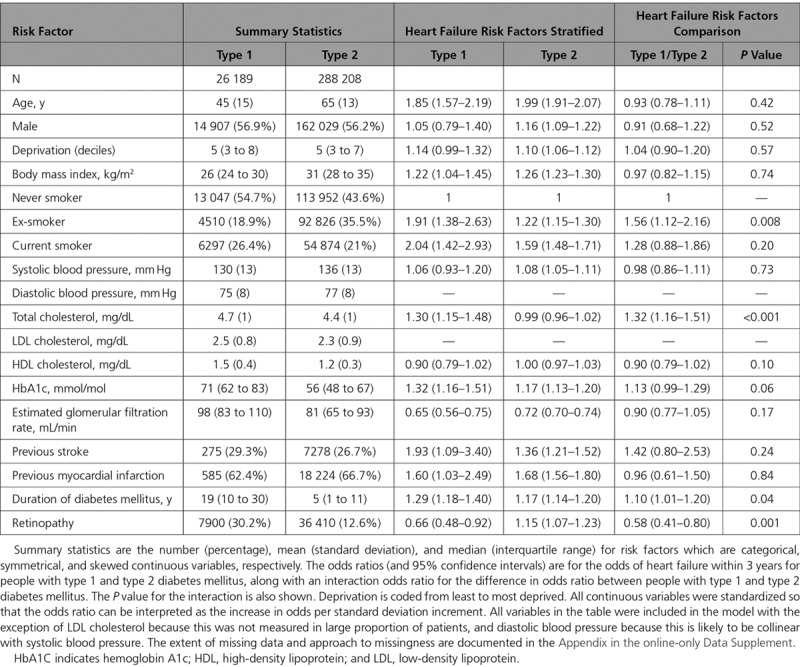
Risk Factors for Heart Failure in People With Diabetes Mellitus: Summary Statistics, Association With Incident Heart Failure Hospitalization Within Three Years, and Comparison of Association in Type 1 Versus Type 2 Diabetes Mellitus

People with type 2 diabetes mellitus were more likely than those with type 1 diabetes mellitus to have a current prescription for a range of cardiovascular drugs (Table 4). Across all drug-classes examined, compared with people with type 1 diabetes mellitus, those with type 2 diabetes mellitus were more likely to have been prescribed cardiovascular medications. For loop diuretics and antiplatelets the risk was not higher after adjusting for age and sex (OR, 0.87; 95% CI, 0.82 to 0.93 and OR, 1.03; 95% CI, 0.99 to 1.07, respectively). However, for the remaining cardiovascular drug classes (including lipid lowering drugs, drugs acting on the renin-angiotensin system and calcium channel blockers) higher prescription levels in type 2 diabetes mellitus persisted on adjusting for age and sex, and on adjusting for the predicted risk of heart failure (Table 4).

**Table 4.**
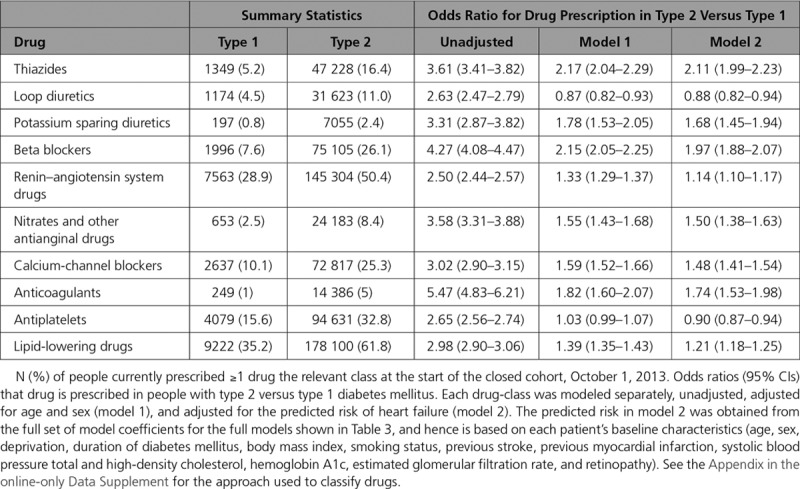
Cardiovascular Drugs in People With Diabetes Mellitus

### Sensitivity Analyses

On restricting incident heart failure admissions to those where heart failure was the primary diagnosis, the heart failure incidence rates were around half of those reported for any incident admission, with rates per 1000 person-years (py) of 0.9 (34 893 events/38 080 361.9 py), 6.1 (11 551 events/1 885 715.8 py), and 2.7 (635 events/237 683.5 py) for the no-diabetes mellitus, type 2 diabetes mellitus, and type 1 diabetes mellitus groups respectively. Nonetheless, compared with the main analysis (Figure 1, Figures IV and V in the online-only Data Supplement, and Table VII in the online-only Data Supplement) similar associations were found (Figures VIII and IX in the online-only Data Supplement and Tables XVII–XIX in the online-only Data Supplement). On adding cardiomyopathy codes (I42.0, I42.7, I42.8, I42.9) to the heart failure definition used in the main analysis, similar results were also found; this was true for the absolute incident rates and case-fatality, and the rate ratios and odds ratio comparing the 3 groups (type 1, type 2 and no diabetes mellitus).

On comparing the clinician-recorded and corrected clinician-recorded definitions of diabetes mellitus type in the closed cohort, similar associations between type of diabetes mellitus and 3-year risk of heart failure were found; adjusting for age, sex, and deprivation, the ORs were 1.24 (95% CI, 1.09–1.40) and 1.21 (95% CI, 1.05–1.38), respectively.

## Discussion

We documented the rate of incident heart failure hospitalizations in a large and complete national dataset which included >250 000 people with diabetes mellitus among a population of >3.25 million people aged ≥30 years, in whom there were >115 000 first hospitalizations for heart failure.

We examined trends in these rates, and the associated 30-day case-fatality, over a 10-year period (2004–2013). Importantly, we reported these rates and trends separately for people with type 1 and type 2 diabetes mellitus.

We found that the age- and sex-adjusted rates of incident heart failure hospitalization were ≈2-fold higher in people with diabetes mellitus, regardless of type, compared with those without diabetes mellitus. In all groups, there was a decline in incidence rate of ≈2% over the decade studied, with a slightly greater rate of decline in people with type 1 diabetes mellitus than in the other groups studied.

In terms of trends, heart failure incident hospitalization appears to be falling more quickly over time (both in absolute and proportional terms) in people with type 1 diabetes mellitus, compared with people with type 2 diabetes mellitus and with people without diabetes mellitus. The difference was moderate; having adjusted for age, sex, and deprivation, the decline was faster in those with type 1 diabetes mellitus (2.1% per year versus 0.2% per year). We are not aware that this finding has been reported previously, but it is consistent with an observation from the Swedish registry that the rate ratio for type 1 diabetes mellitus and incident heart failure was larger in the 1998 to 2004 period than in the 2005 to 2011 period.^2^

We also found that the relative difference in heart failure incidence between people without diabetes mellitus and those with type 1 or type 2 diabetes mellitus was larger for younger people and for women than for older people and men. This is consistent with previous reports.^1–3^ It is important to note, however, that because of the higher overall rates in men and older people, the absolute differences in heart failure incidence were larger in these groups. Therefore, from the perspective of the individual patient, the impact of diabetes mellitus on heart failure risk is greater at older ages and in men. Indeed, the absolute difference in 1-year risk of heart failure admission in 80-year-old men (the group with the smallest relative difference) with and without diabetes mellitus was 2.5%. For the same comparison, but in 40-year-old women, the difference was <0.5%.

Unlike incidence, for case-fatality the relationship with diabetes mellitus depended on the type of diabetes mellitus. Case-fatality was similar among people with type 2 diabetes mellitus compared with those without diabetes mellitus. However, among people with type 1 diabetes mellitus, case-fatality was 1.3-fold higher in women and 1.9-fold higher in men.

Women therefore have a 2-fold higher heart failure incidence and a 1.3-fold higher case-fatality. In combination, this means that compared with women without diabetes mellitus, women with type 1 diabetes mellitus have around a 2.5-fold higher risk of having an incident heart failure admission which results in death within 30 days. For the equivalent comparison in men, there is almost a 4-fold difference in risk.

Elevated incidence rates for heart failure admission among people with type 2 diabetes mellitus have been reported previously. One study using the Clinical Practice Research Database (CPRD) used primary care records to define heart failure and type 2 diabetes mellitus. The authors identified 34 198 people with type 2 diabetes mellitus from 1998 to 2010. Among women >60 years of age with and without type 2 diabetes mellitus, they found a relative difference (hazard ratio) of 1.50.^3^ We obtained a similar relative difference (rate ratio) of 2.12 (95% CI, 2.06–2.17). For men >60 years of age, the comparable figures were 1.43 and 1.93 (95% CI, 1.88–1.98), respectively. The fact that we included only hospitalizations, and not primary care attendances, may account for the slightly stronger associations we report.

In the international Reduction of Atherothrombosis for Continued Health (REACH) registry, there was also an elevated risk of heart failure hospitalization among the 19 699 people with diabetes mellitus (type unspecified) compared to people without diabetes mellitus.^1^ However, the magnitude of the association in this cohort, which mainly included people with established atherothrombotic disease or risk factors for atherothrombosis, was weaker than we found at 1.33-fold. In sensitivity analyses excluding any patient with a previous ischemic heart disease admission, we continued to observe an ≈2-fold association. However, we did not have any measures of stable coronary disease. As such, one explanation for the stronger association we observed is that the increased heart failure risk in diabetes mellitus is partly related explained through increased risk of atheromatous disease.

Previous studies examining case-fatality after heart failure admissions among people with type 2 diabetes mellitus have been equivocal. One multi-center register study found no difference in in-hospital mortality among people with diabetes mellitus compared to those without (OR, 1.00; 95% CI, 0.88–1.14).^12^ Similarly, in a study in the Scottish population comparing incident heart failure admissions from 1986 to 2003, the age–sex adjusted 30-day case-fatality following heart failure admission was lower in people with diabetes mellitus than in those without diabetes mellitus (for example in men aged 65–74 the OR was 0.82; 95% CI, 0.73–0.93), although case-fatality at 1 year was higher.^13^ Neither study, however, included data on diabetes mellitus type. Because the majority of people with diabetes mellitus have type 2 diabetes mellitus, these reports are consistent with our own finding that people with type 2 diabetes mellitus did not have a higher case-fatality than individuals without diabetes mellitus.

Fewer studies have examined heart failure as a complication of type 1 diabetes mellitus. The largest, which was a population-based study from Sweden, and which updates and extends a previous report from the same diabetes mellitus registry,^14^ reported a rate of incident heart failure hospitalization of 6.7 per 1000 person-years in people with type 1 diabetes mellitus compared to 4.0 per 1000 person-years in individuals without diabetes mellitus, similar to the difference we observed.^2^ However, those investigators did not examine trends over time or case-fatality. Indeed, ours is the first population-based study of which we are aware to compare case-fatality after heart failure admissions in people with and without type 1 diabetes mellitus, and we are not aware that the high case-fatality in patients with type 1 diabetes mellitus has been reported previously.

The mechanisms underlying this difference in case-fatality are unknown, and mechanistic studies are needed. Indeed, excepting coronary artery atherosclerosis, the mechanisms underlying the relationship between diabetes mellitus and heart failure are not well understood, and whether or not diabetic cardiomyopathy represents a distinct entity remains controversial.^15–17^ Notwithstanding the mechanisms, however, the increased case-fatality provides additional support for the view that heart failure is an under-recognized and important complication in type 1 diabetes mellitus.^18^

Time trends in case-fatality were similar across groups. For people with and without diabetes mellitus, regardless of type, we found that case-fatality fell by around 3% per-year. We were unable to identify any previous study which compared heart failure admission case-fatality trends among people with and without diabetes mellitus. Nonetheless, similar trends have been reported in unselected patients, and in people with type 2 diabetes mellitus. In terms of the former, a study using the Swedish national hospital discharge register, which included 13.6% of people with type 2 diabetes mellitus, found a decline of ≈4% per-year (hazard ratio 0.96 per year during the 5-year period from 1987–2006).^19^ Also, trends after heart failure admissions were examined for people with type 2 diabetes mellitus in a subset of the U.S. National Inpatient Sample mortality (defined using administrative data – ICD-9-CM codes 250.0–250.9 with a fifth digit of 0 or 2). In models adjusting for age and sex, case-fatality fell by ≈5% per year from 2000 to 2010.^20^ This is consistent, therefore, with our findings.

In the general adult population, a number of risk factors such as age, sex, deprivation, smoking, obesity, hypertension, cholesterol, and previous cardiovascular disease are known to predict heart failure.^21^ Lind et al^14^ also showed that, among people with type 1 diabetes mellitus, smoking, systolic blood pressure, high body mass index, duration of diabetes mellitus, and hemoglobin A1c were associated with increased heart failure incidence. We obtained similar findings for the association between these risk factors and the 3-year risk of heart failure among people with type 1 diabetes mellitus. We have also been able to show that very similar associations (both in terms of magnitude and direction) are evident for people with type 2 as well as type 1 diabetes mellitus.

We also found that people with type 1 diabetes mellitus were less commonly prescribed drugs known to reduce the risk of heart failure (whether directly or through reducing the risk of heart disease); this included antihypertensives, drugs acting on the renin–angiotensin, system and lipid-lowering drugs.^22^ Importantly, these differences were still found after taking into account the fact that people with type 2 diabetes mellitus are older, and even after adjusting for each patient’s predicted risk of heart failure (based on their baseline characteristics). Considerable caution is needed in interpreting this finding, as the clinical risk factors (blood pressure, smoking, etc) used to adjust for baseline risk were obtained as part of routine clinical care, not in a prospective study, and were not available for some patients. Nonetheless, this observation does raise the possibility that, even where the heart failure risk is similar, people with type 1 diabetes mellitus may be less likely than people with type 2 diabetes mellitus to receive preventative drug therapy.

The strengths of our study include the very large population-based nature of the electronic record of diagnosed diabetes mellitus that captures data for >99% of the population of Scotland, information on the type of diabetes mellitus, and the availability of linkage to quality-assured hospital admission and mortality data for the whole population.^23^ A limitation is that heart failure events insufficiently severe to require admission to hospital were not captured as we did not have access to primary care data.

However, we have previously shown via review by 2 independent clinicians (a generalist and cardiologist) that heart failure admissions in the Scottish hospitalization database are reliably recorded.^24^ Moreover, similar associations were found in a sensitivity analysis which restricted the definition of heart failure to admission where this was coded in the first diagnostic position.

Secondly, although the data were of high quality, some misclassification as a result of diagnostic uncertainty is likely to have occurred. In particular, some people with type 1 diabetes mellitus will have been classified as type 2 diabetes mellitus, and vice versa. However, there is no reason to suppose that this misclassification will have been differential by outcome, and it is therefore likely to have attenuated any observed differences between type 1 and type 2 diabetes mellitus. Indeed, in the cohort restricted to patients with diabetes mellitus in 2013, the use of a more precise definition of diabetes mellitus type did not importantly affect the estimated 3-year risk of diabetes mellitus. A further limitation is that we did not have accurate data on race or ethnicity, and so we cannot comment on whether associations between diabetes mellitus and heart failure incidence differ by these variables.

Finally, we did not have access to sufficient data on left ventricular function to comment on the relative contribution of reduced and preserved ejection fraction heart failure on the differences between people with and without diabetes mellitus.

### Conclusion

Despite falling incidence rates, particularly in type 1 diabetes mellitus, heart failure remains ≈2-fold higher than in people without diabetes mellitus, with case-fatality also being higher in people with type 1 diabetes mellitus. Those with type 1 diabetes mellitus also received fewer preventative cardiovascular medications. These findings provide additional support for the view that heart failure is an under-recognized and important complication in diabetes mellitus, particularly among people with type 1 diabetes mellitus.

## Sources of Funding

Dr McAllister is funded via an Intermediate Clinical Fellowship and Beit Fellowship from the Wellcome Trust (201492/Z/16/Z).

## Disclosures

Dr Jhund reports grants from Boehringer Ingelheim, during the conduct of the study, and personal fees from Novartis, Cytokinetics, and Visor Pharma, outside the submitted work. Dr Petrie reports personal fees and other from Novo Nordisk, grants and personal fees from Sanofi Aventis, nonfinancial support from Merck (Germany), personal fees from Lilly, nonfinancial support from Itamar Medical, grants and personal fees from Quintiles, grants and personal fees from Janssen, personal fees from ACI Clinical, and personal fees from Pfizer, outside the submitted work. Dr Sattar’s employer received funding from Boehringer Ingelheim for a research grant, and Dr Sattar has received personal speaking fees from Boehringer Ingelheim, Novo Nordisk, Eli Lilly, and Roche Diagnostics and has acted as a consultant for Boehringer Ingelheim, Janssen, and Astrazeneca. Dr McMurray’s employer, Glasgow University, is currently or has recently been paid for his role in trials testing treatments in patients with diabetes mellitus: principal investigator DAPA-HF trial dapagliflozin AstraZeneca, principal investigator BEST trial bexagliflozin Theracos, coprincipal Investigator HARMONY Outcomes trial albiglutide GSK, executive committee member DAPA-CKD trial dapagliflozin AstraZeneca, executive committee member ACE trial acarbose Oxford University/Bayer, executive committee member SONAR trial atrasentan Abbvie, and data monitoring committee member EXSCEL trial exenatide AstraZeneca. Dr McMurray’s study sponsors have paid for his travel and accommodation for some meetings related to these trials. The remaining authors report no conflicts.

## Supplementary Material
