# Supplementary material for: Incidence of Hospitalization for Heart Failure and Case-Fatality Among 3.25 Million People With and Without Diabetes Mellitus
Source: Circulation. 2018 Dec 10;138(24):2774–86. doi: 10.1161/CIRCULATIONAHA.118.034986 (PMC6287897; doi:10.1161/CIRCULATIONAHA.118.034986)
Supplement: Supplementary file 1 [file cir-138-2774-s001.pdf]

SUPPLEMENTAL MATERIAL

Incidence of hospitalisation for heart failure and case-fatality among 3.25 million people with and without diabetes

David A McAllister MD, Stephanie Read PhD, Jan Kerssens PhD, Shona Livingstone MSc, Stuart McGurnaghan, Pardeep Jhund PhD, John Petrie PhD, Naveed Sattar PhD, Colin Fischbacher MD, Soren Lund Kristensen PhD, John McMurray PhD, Helen M Colhoun PhD and Sarah Wild PhD**

Contents

Aggregated data **2**

Table S1 Heart failure admissions by age, sex, deprivation and diagnosis 2

Table S2 Heart failure deaths within 30 days of admission by age, sex, deprivation and diagnosis . 3

Classification of drugs **5**

Table S3 Prescribed drugs, groups each BNF heading assigned to 5

Incidence rate calculation using a look-back period **6**

Figure S1 Admissions and incident events in 3 example patients 6

Table S4 components of person time calculation 6

Table S5 person time and rate calculation 7

Missing data and imputation for risk factor data **8**

Table S6 Proportion of missing data for each variable for the 2013 cohort of people with type 1 and type 2 diabetes, used to estimate associations with clinical risk factors 8

Main analysis - additional tables and figures **9**

Figure S2 Prevalence of heart failure by diabetes type, age and sex 9

Figure S3 Prevalence of heart failure by diabetes type, deprivation and sex 10

Table S7 Cross-sectional prevalence model, coefficients and standard errors 10

Figure S4 Incidence rate of heart failure by diabetes type, deprivation and sex 11

Table S8 Cross-sectional incidence rate model, coefficients and standard errors 11

Figure S5 Modelled rate of heart failure by diabetes type, deprivation and sex. Rates shown on absolute scale 12

Table S9 Time trends incidence rate model, coefficients and standard errors 12

Table S10 Estimates of non-parametric smooth functions 13

Table S11, Heart failure case-fatality model, coefficients and standard errors 13

Table S12, Heart failure case-fatality over time model, coefficients and standard errors 14

Additional analysis - IHD admissions excluded 14

Table S13 Heart failure admissions and deaths by age, sex, deprivation and diagnosis with IHD admissions excluded . . . 14

Figure S6 Modelled rate of heart failure by diabetes type, age and sex with IHD admissions excluded 16

Table S14 Cross-sectional model, coefficients and standard errors with IHD admissions excluded . 16

Figure S7 Trends in rates of heart failure by diabetes type, sex and calendar year with IHD admissions excluded . . . 17

Table S15 Time trends model, coefficients and standard errors with IHD admissions excluded . . . 17

Table S16 Estimates of non-parametric smooth functions with IHD admissions excluded 18

Sensitivity analysis - Events coded in First position only 19

Figure S8 Modelled rate of heart failure by diabetes type, age and sex with diagnosis recored in first position only . . . 19

Table S17 Cross-sectional model, coefficients and standard errors with diagnosis recored in first position only . . . 19

Figure S9 Trends in rates of heart failure by diabetes type, sex and calendar year with with diagnosis recored in first position only . . . 20

Table S18 Time trends model, coefficients and standard errors with diagnosis recored in first position only . . . 20

Table S19 Estimates of non-parametric smooth functions with with diagnosis recored in first position only . . . 21

Legend for interactive figure . . . 21

References 22

Aggregated data

Table S1 Heart failure admissions by age, sex, deprivation and diagnosis

Age	Sex	Deprivation	Diagnosis	Admissions	Persontime
20 to 49	men	1-5	No diabetes	1087	5069751
20 to 49	men	1-5	Type1	40	41394
20 to 49	men	1-5	Type 2	86	49255
20 to 49	men	6-10	No diabetes	2065	5263772
20 to 49	men	6-10	Type1	77	44487
20 to 49	men	6-10	Type 2	176	76730
20 to 49	women	1-5	No diabetes	433	5249525
20 to 49	women	1-5	Type1	29	30875
20 to 49	women	1-5	Type 2	31	31915
20 to 49	women	6-10	No diabetes	945	5601676
20 to 49	women	6-10	Type1	46	32760
20 to 49	women	6-10	Type 2	103	58611
50 to 59	men	1-5	No diabetes	2055	1693189
50 to 59	men	1-5	Type1	46	12610
50 to 59	men	1-5	Type 2	385	99307

Age	Sex	Deprivation	Diagnosis	Admissions	Persontime
50 to 59	men	6-10	No diabetes	2955	1458296
50 to 59	men	6-10	Type1	98	11905
50 to 59	men	6-10	Type 2	708	123769
50 to 59	women	1-5	No diabetes	704	1794524
50 to 59	women	1-5	Type1	39	8931
50 to 59	women	1-5	Type 2	145	56955
50 to 59	women	6-10	No diabetes	1416	1569962
50 to 59	women	6-10	Type1	69	8380
50 to 59	women	6-10	Type 2	303	87522
60 to 69	men	1-5	No diabetes	3812	1263936
60 to 69	men	1-5	Type1	75	7191
60 to 69	men	1-5	Type 2	1175	145099
60 to 69	men	6-10	No diabetes	5353	1124748
60 to 69	men	6-10	Type1	97	6804
60 to 69	men	6-10	Type 2	1883	163745
60 to 69	women	1-5	No diabetes	1865	1411898
60 to 69	women	1-5	Type1	49	6010
60 to 69	women	1-5	Type 2	500	92678
60 to 69	women	6-10	No diabetes	3257	1287385
60 to 69	women	6-10	Type1	79	5690
60 to 69	women	6-10	Type 2	1134	132674
70 to 79	men	1-5	No diabetes	5934	741182
70 to 79	men	1-5	Type1	87	3469
70 to 79	men	1-5	Type 2	2121	121589
70 to 79	men	6-10	No diabetes	7511	704085
70 to 79	men	6-10	Type1	114	3372
70 to 79	men	6-10	Type 2	2743	135025
70 to 79	women	1-5	No diabetes	4479	943917
70 to 79	women	1-5	Type1	82	3766
70 to 79	women	1-5	Type 2	1383	103576
70 to 79	women	6-10	No diabetes	7232	987117
70 to 79	women	6-10	Type1	102	3962
70 to 79	women	6-10	Type 2	2445	142301
80 to 89	men	1-5	No diabetes	7690	339174
80 to 89	men	1-5	Type1	35	899
80 to 89	men	1-5	Type 2	1737	49383
80 to 89	men	6-10	No diabetes	7878	314140
80 to 89	men	6-10	Type1	38	833
80 to 89	men	6-10	Type 2	1717	46507
80 to 89	women	1-5	No diabetes	11513	640946
80 to 89	women	1-5	Type1	48	1251
80 to 89	women	1-5	Type 2	1843	64392
80 to 89	women	6-10	No diabetes	13245	653518
80 to 89	women	6-10	Type1	63	1334
80 to 89	women	6-10	Type 2	2341	74249

Numbers less than or equal to 5 were suppressed to maintain confidentiality.

Table S2 Heart failure deaths within 30 days of admission by age, sex, deprivation and diagnosis

Age	Sex	Deprivation	Diagnosis	Admissions	Deaths
20 to 29	men	1-5	pop	75	<=5
20 to 29	men	1-5	t1dm	<=5	<=5
20 to 29	men	1-5	t2dm	<=5	<=5
20 to 29	men	6-10	pop	124	<=5
20 to 29	men	6-10	t1dm	6	<=5
20 to 29	men	6-10	t2dm	<=5	<=5
20 to 29	women	1-5	pop	51	<=5
20 to 29	women	1-5	t1dm	<=5	<=5
20 to 29	women	6-10	pop	79	<=5
20 to 29	women	6-10	t1dm	<=5	<=5
20 to 29	women	6-10	t2dm	<=5	<=5
30 to 49	men	1-5	pop	1012	31
30 to 49	men	1-5	t1dm	36	6
30 to 49	men	1-5	t2dm	85	<=5
30 to 49	men	6-10	pop	1941	100
30 to 49	men	6-10	t1dm	71	<=5
30 to 49	men	6-10	t2dm	173	<=5
30 to 49	women	1-5	pop	382	26
30 to 49	women	1-5	t1dm	27	<=5
30 to 49	women	1-5	t2dm	31	<=5
30 to 49	women	6-10	pop	866	71
30 to 49	women	6-10	t1dm	41	<=5
30 to 49	women	6-10	t2dm	98	6
50 to 59	men	1-5	pop	2055	76
50 to 59	men	1-5	t1dm	46	<=5
50 to 59	men	1-5	t2dm	385	23
50 to 59	men	6-10	pop	2955	181
50 to 59	men	6-10	t1dm	98	10
50 to 59	men	6-10	t2dm	708	46
50 to 59	women	1-5	pop	704	30
50 to 59	women	1-5	t1dm	39	<=5
50 to 59	women	1-5	t2dm	145	10
50 to 59	women	6-10	pop	1416	126
50 to 59	women	6-10	t1dm	69	7
50 to 59	women	6-10	t2dm	303	28
60 to 69	men	1-5	pop	3812	264
60 to 69	men	1-5	t1dm	75	14
60 to 69	men	1-5	t2dm	1175	76
60 to 69	men	6-10	pop	5353	453
60 to 69	men	6-10	t1dm	97	15
60 to 69	men	6-10	t2dm	1883	160
60 to 69	women	1-5	pop	1865	175
60 to 69	women	1-5	t1dm	49	<=5
60 to 69	women	1-5	t2dm	500	39
60 to 69	women	6-10	pop	3257	390
60 to 69	women	6-10	t1dm	79	10
60 to 69	women	6-10	t2dm	1134	119
70 to 79	men	1-5	pop	5934	684
70 to 79	men	1-5	t1dm	87	17
70 to 79	men	1-5	t2dm	2121	261
70 to 79	men	6-10	pop	7511	1001
70 to 79	men	6-10	t1dm	114	23

Age	Sex	Deprivation	Diagnosis	Admissions	Deaths
70 to 79	men	6-10	t2dm	2743	326
70 to 79	women	1-5	pop	4479	578
70 to 79	women	1-5	t1dm	82	17
70 to 79	women	1-5	t2dm	1383	166
70 to 79	women	6-10	pop	7232	997
70 to 79	women	6-10	t1dm	102	13
70 to 79	women	6-10	t2dm	2445	321
80 to 89	men	1-5	pop	7690	1531
80 to 89	men	1-5	t1dm	35	10
80 to 89	men	1-5	t2dm	1737	307
80 to 89	men	6-10	pop	7878	1579
80 to 89	men	6-10	t1dm	38	13
80 to 89	men	6-10	t2dm	1717	322
80 to 89	women	1-5	pop	11513	2260
80 to 89	women	1-5	t1dm	48	10
80 to 89	women	1-5	t2dm	1843	350
80 to 89	women	6-10	pop	13245	2627
80 to 89	women	6-10	t1dm	63	14
80 to 89	women	6-10	t2dm	2341	440

Classification of drugs

All drugs for people with diabetes are incldued in the Scottish diabetes register, having been extracted from primary care records. These are assigned to a British National Formulary (BNF) chapter, section and paragraph heading. We collapsed the BNF headings to each of the groups shown in Table S3. See https://openprescribing.net/bnf/ for a complete list of headings. Patients were counted as having been prescribed the drug if they were currently prescribed a drug within that class on the cohort start date, the 1st of October 2013.

Table S3 Prescribed drugs, groups each BNF heading assigned to

BNF heading	BNF heading label	Group
2.2.1	Thiazides And Related Diuretics	Thiazides
2.2.2	Loop Diuretics	Loop
2.2.3	Pot-Sparing Diuretics&Aldosterone Antag	Potassium sparing
2.2.4	Potassium Sparing Diuretics & Compounds	Potassium sparing
2.4	Beta-Adrenoceptor Blocking Drugs	Beta blockers
2.5.5	Renin-Angiotensin System Drugs	Renin-Angiotensin System Drugs
2.6.1	Nitrates	Nitrates and other anti-anginal
2.6.2	Calcium-Channel Blockers	Calcium-Channel Blockers
2.6.3	Other Antianginal Drugs	Nitrates and other anti-anginal
2.8.2	Oral Anticoagulants	Anticoagulants
2.9	Antiplatelet Drugs	Antiplatelets
2.12	Lipid-Regulating Drugs	Lipid lowering drugs

Incidence rate calculation using a look-back period

Figure S1 shows a worked example for the calculation of events and person-time for a notional population stratum. For example, men born in 1968 who did not have diabetes. In this example, 3 people had one or more admission with heart failure during the follow-up period, and/or during the 10 year look-back period. The calculation of year-specific incident counts is straightforward, and is shown alongside the figure. Any event after the start of the cohort period, where there was no previous event within 10 years is considered incident and this is summed across rows.

The person-years calculation is more complex and is shown in Tables S4 and S5.

Figure S1 Admissions and incident events in 3 example patients

	One		Two		Three		Total
	A	I	A	I	A	I	I
1994							
1995	1	0					
1996							
1997							
1998							
1999							
2000							
2001							
2002							
2003					1	0	
2004							0
2005							0
2006							0
2007							0
2008							0
2009							0
2010							0
2011	1	1	1	1			2
2012							0
2013							0

A - admission, I incident event.

Table S4 components of person time calculation

Year	POP	DM	p1	p2	p3
2004	190	10	0	1	0
2005	190	10	0	1	0
2006	190	10	1	1	0
2007	190	10	1	1	0
2008	190	10	1	1	0
2009	190	12	1	1	0
2010	190	12	1	1	0
2011	185	12	0	0	0
2012	185	12	0	0	0
2013	185	13	0	0	1

POP refers to the mid-year estimate for the population (based on National Records Scotland census data and mid-year estimation modelling). DM refers to the population with diabetes (from the diabetes register) and p1, p2 and p3 refers to the person-time for the 3 patients.

Since patient 1 had an admission in 1995, they were not eligible to have another incident event within ten years, and so the person-time for each of these periods is removed. Patient 2 had an incident event in 2011 and so only contributed 7 person-years. Patient 3 had an event in 2003 which was not incident, and as a consequence contributed only one person year.

The person-time for each year, within each stratum, is calculated as follows:-

$$PT = POP - DM - N + p_1 + p_2 + p_3 + ...p_n$$

Where POP, DM and p_n are as per Table S4 and N indicates the number of patients with events observed.

In R, for this example, this would be calculated as follows, along with the calendar-year/stratum-specific rate.

```
pt_ill2 <- pt_ill %>%
  mutate(`Person time` = POP - DM - 3 + p1 + p2 + p3,
         `Rate per 1000 person-years` = 1000* Incident/`Person time`)
```

Table S5 person time and rate calculation

Year	POP	DM	p1	p2	p3	Incident	Person time	Rate per 1000 person-years
2004	190	10	0	1	0	0	178	0.0
2005	190	10	0	1	0	0	178	0.0
2006	190	10	1	1	0	0	179	0.0
2007	190	10	1	1	0	0	179	0.0
2008	190	10	1	1	0	0	179	0.0
2009	190	12	1	1	0	0	177	0.0
2010	190	12	1	1	0	0	177	0.0
2011	185	12	0	0	0	2	170	11.8
2012	185	12	0	0	0	0	170	0.0
2013	185	13	0	0	1	0	170	0.0

Missing data and imputation for risk factor data

For the cohort with diabetes identified in 2013, there was missing data for a number of variables (Table S6).

Table S6 Proportion of missing data for each variable for the 2013 cohort of people with type 1 and type 2 diabetes, used to estimate associations with clinical risk factors

	Type 2	Type 1
Age	0%	0%
BMI	20.1%	16.4%
Deprivation (Deciles)	0%	0%
Diastolic BP	7.5%	9.1%
EGFR	39.8%	42.5%
HbA1	17.6%	11.2%
HDL	18.8%	19.2%
LDL	71.1%	57.9%
Retinopathy	0%	0%
Sex	0%	0%
Smoking status	9.2%	8.9%
Systolic BP	7.5%	9.1%
Total cholesterol	11.1%	13%

Summary statistics for each variable are reported (in Table 3 in the main manuscript) based on the available data for each variable. For the logistic regression models, we carried out multiple imputation, using the MICE package in R.[1]

We obtained 5 imputed datasets, using all the variables included in the planned model as well as LDL cholesterol and diastolic blood pressure. Imputation was performed using the following methods for each variable-type:- predictive mean matching for continuous variables, logistic regression for binary variables, polytomous regression for unordered categorical variables and proportional odds model for ordered categorical variables.

We then fit a logistic regression model to each imputed dataset and pooled the results using the method described by Barnard and Rubin.[2] Results of the modelling are shown in Table 3 of the main manscript.

Main analysis - additional tables and figures

Figure S2 Prevalence of heart failure by diabetes type, age and sex

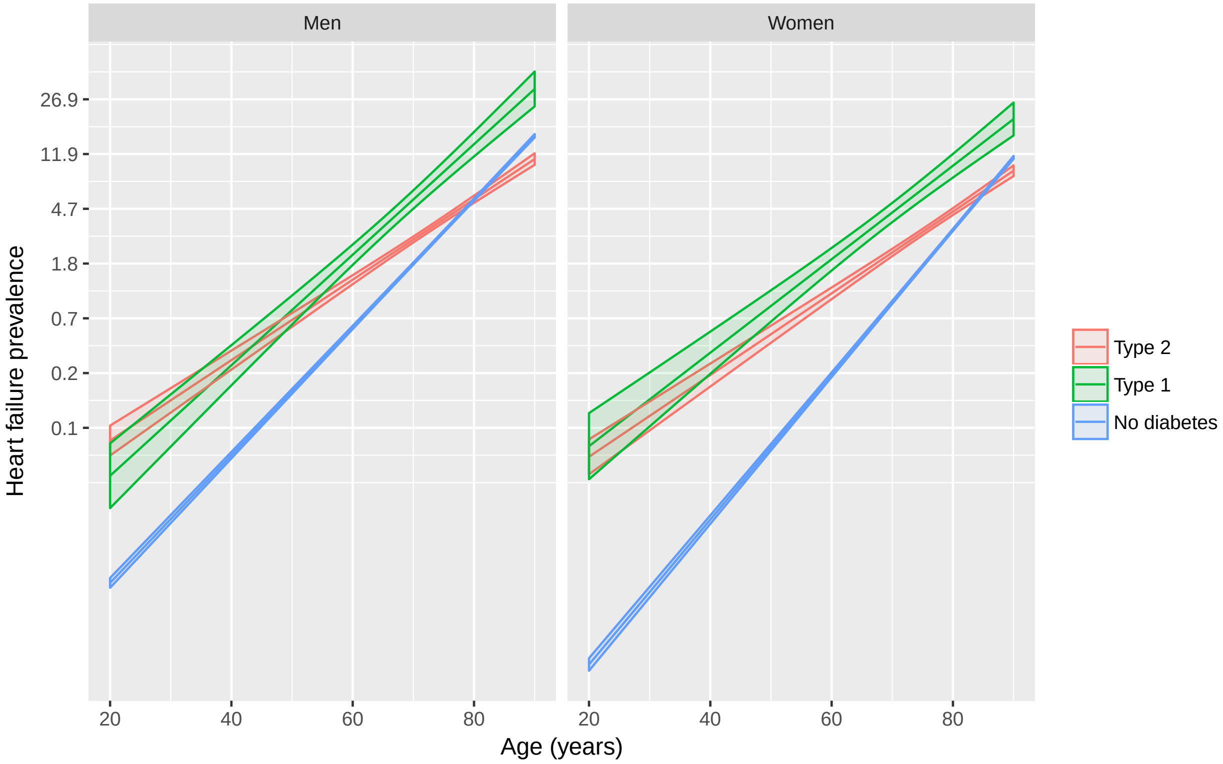

The lines represent the predicted prevalences obtained from logistic regression models of prevalent heart failure events on age, sex, deprivation and diabetes type, with interaction terms included where these improved model fit. Predictions were made at the median deprivation score.

Figure S3 Prevalence of heart failure by diabetes type, deprivation and sex

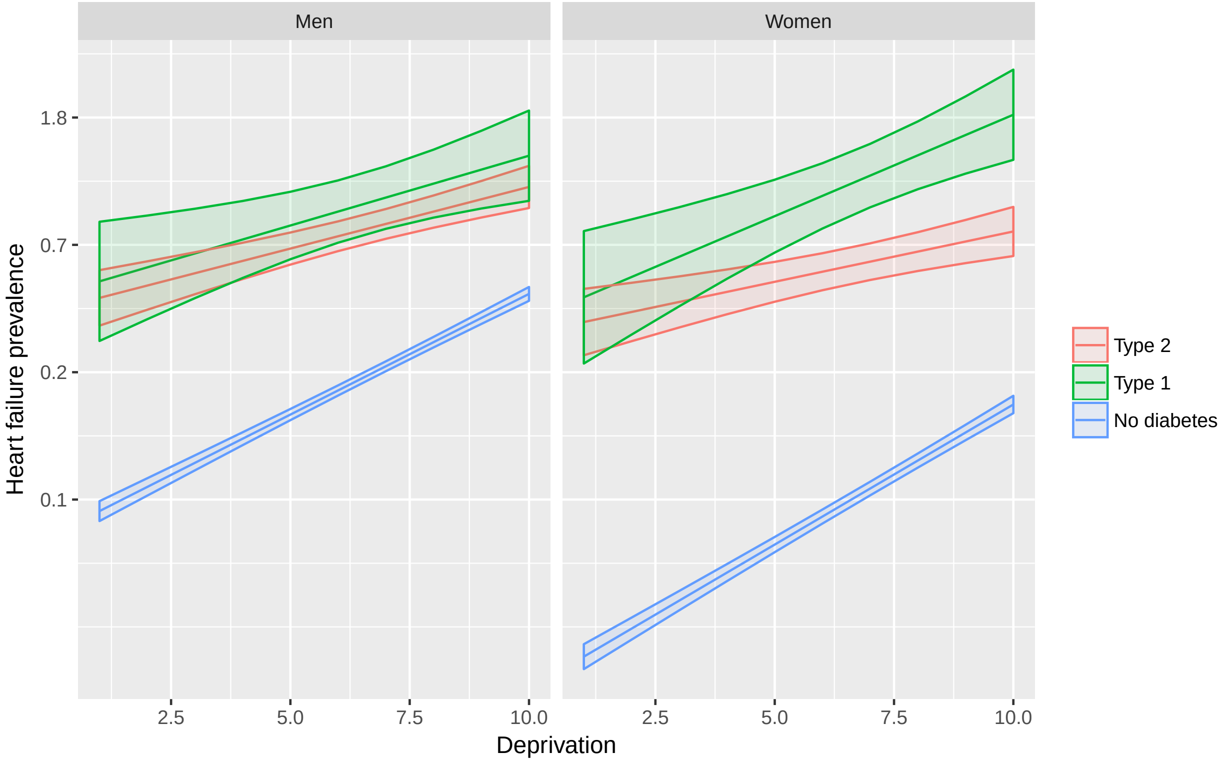

The lines represent the predicted prevalences obtained from logistic regression models of prevalent heart failure events on age, sex, deprivation and diabetes type, with interaction terms included where these improved model fit. Predictions were made at age 50.

Table S7 Cross-sectional prevalence model, coefficients and standard errors

	Estimate	Std. Error	t value	Pr(>|t|)
(Intercept)	-7.28	0.045	-161.023	<0.001
age_ten	1.371	0.016	87.729	<0.001
sex	-1.173	0.048	-24.383	<0.001
dep_two	0.948	0.03	31.527	<0.001
typet1dm	1.884	0.275	6.846	<0.001
typet2dm	1.767	0.134	13.194	<0.001
age_ten:sex	0.156	0.012	13.405	<0.001
age_ten:dep_two	-0.205	0.01	-19.533	<0.001
sex:dep_two	0.153	0.024	6.32	<0.001
age_ten:typet1dm	-0.196	0.109	-1.788	0.074
age_ten:typet2dm	-0.57	0.051	-11.209	<0.001
sex:typet1dm	0.999	0.327	3.058	0.002
sex:typet2dm	1.001	0.142	7.052	<0.001
dep_two:typet1dm	-0.399	0.189	-2.11	0.035
dep_two:typet2dm	-0.464	0.091	-5.093	<0.001
age_ten:sex:typet1dm	-0.312	0.081	-3.835	<0.001
age_ten:sex:typet2dm	-0.145	0.039	-3.747	<0.001
age_ten:dep_two:typet1dm	0.039	0.074	0.523	0.601

	Estimate	Std. Error	t value	Pr(>|t|)
age_ten:dep_two:typet2dm	0.138	0.035	3.949	<0.001
sex:dep_two:typet1dm	0.096	0.205	0.467	0.641
sex:dep_two:typet2dm	-0.241	0.073	-3.325	0.001

Logistic regression model with admission as the outcome. Age_ten is the age in years divided by ten and dep_two is the deprivation score divided by two.

Figure S4 Incidence rate of heart failure by diabetes type, deprivation and sex

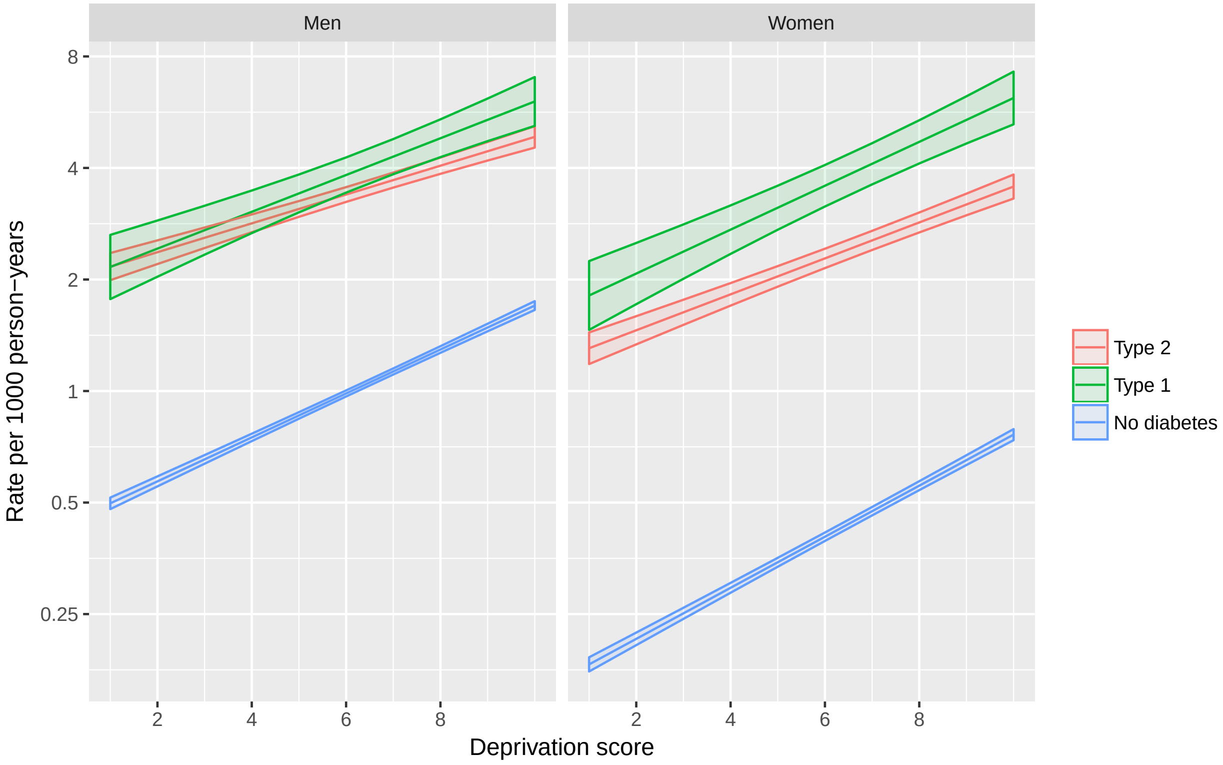

The lines represent the predicted rates obtained from quasi-Poisson regression models of incident heart failure events on age, sex, deprivation and diabetes type, with interaction terms included where these improved model fit. Predictions were made for men and women aged 50.

Table S8 Cross-sectional incidence rate model, coefficients and standard errors

	Estimate	Std. Error	t value	Pr(>|t|)
(Intercept)	-7.742	0.021	-372.393	<0.001
age_ten	1.131	0.007	154.279	<0.001
sex	-1.023	0.023	-45.447	<0.001
dep_two	0.683	0.014	47.613	<0.001
typet1dm	1.491	0.117	12.765	<0.001
typet2dm	1.518	0.053	28.734	<0.001
age_ten:sex	0.162	0.006	28.1	<0.001
age_ten:dep_two	-0.167	0.005	-32.915	<0.001

	Estimate	Std. Error	t value	Pr(>|t|)
sex:dep_two	0.11	0.012	9.364	<0.001
age_ten:typet1dm	-0.177	0.054	-3.294	0.001
age_ten:typet2dm	-0.289	0.02	-14.751	<0.001
sex:typet1dm	0.824	0.087	9.479	<0.001
sex:typet2dm	0.494	0.042	11.852	<0.001
dep_two:typet1dm	-0.111	0.079	-1.413	0.158
dep_two:typet2dm	-0.235	0.037	-6.433	<0.001
age_ten:sex:typet1dm	-0.204	0.041	-5.013	<0.001
age_ten:sex:typet2dm	-0.114	0.015	-7.363	<0.001
age_ten:dep_two:typet1dm	0.047	0.036	1.301	0.193
age_ten:dep_two:typet2dm	0.053	0.014	3.919	<0.001

Quasi-Poisson regression model with admissions or death as the outcome. Age_ten is the age in years divided by ten and dep_two is the deprivation score divided by two. The standard errors and P-values are scaled to allow for overdispersion.

Figure S5 Modelled rate of heart failure by diabetes type, deprivation and sex. Rates shown on absolute scale

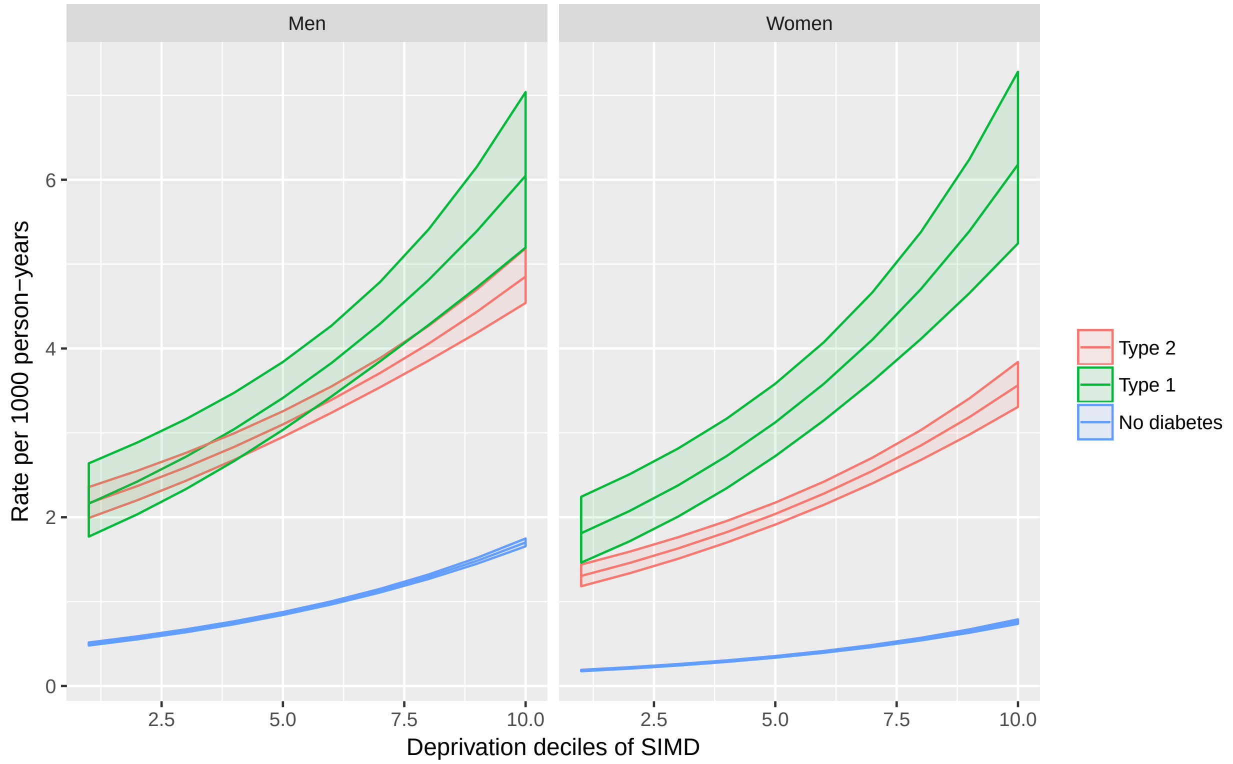

This figure is is simiar to Figure one in the main manuscript, using the same regression model, but with deprivation rather than age being shown on the x-axis. The lines represent the predicted rates obtained from quasi-Poisson regression models of incident heart failure events on age, sex, deprivation and diabetes type, with interaction terms included where these improved model fit. Predictions were made at age 50.

Table S9 Time trends incidence rate model, coefficients and standard errors

	Estimate	Std. Error	t value	Pr(>|t|)
(Intercept)	-7.738	0.021	-373.826	<0.001
age_ten	1.135	0.007	155.51	<0.001
sex	-1.024	0.022	-45.789	<0.001
dep_two	0.682	0.014	47.877	<0.001
year	-0.009	0.003	-3.774	<0.001
typet1dm	1.511	0.116	13.031	<0.001
typet2dm	1.521	0.053	28.946	<0.001
age_ten:sex	0.161	0.006	28.112	<0.001
age_ten:dep_two	-0.168	0.005	-33.366	<0.001
sex:dep_two	0.111	0.012	9.485	<0.001
age_ten:year	-0.005	0.001	-5.481	<0.001
age_ten:typet1dm	-0.181	0.053	-3.399	0.001
age_ten:typet2dm	-0.287	0.02	-14.714	<0.001
sex:typet1dm	0.823	0.086	9.549	<0.001
sex:typet2dm	0.493	0.041	11.896	<0.001
dep_two:typet1dm	-0.116	0.078	-1.489	0.137
dep_two:typet2dm	-0.234	0.036	-6.453	<0.001
year:typet1dm	-0.022	0.01	-2.125	0.034
year:typet2dm	-0.004	0.003	-1.481	0.139
age_ten:sex:typet1dm	-0.206	0.04	-5.102	<0.001
age_ten:sex:typet2dm	-0.113	0.015	-7.399	<0.001
age_ten:dep_two:typet1dm	0.047	0.036	1.306	0.192
age_ten:dep_two:typet2dm	0.054	0.014	4.002	<0.001

Quasi-Poisson regression model with admissions or death as the outcome. Age_ten is the age in years divided by ten and dep_two is the deprivation score divided by two. The standard errors and P-values are scaled to allow for overdispersion.

Table S10 Estimates of non-parametric smooth functions

	Estimated degrees of freedom	Chi-squared	Approximate P-value
No diabetes	7.825	453.517	<0.001
Type 1	1.916	17.512	<0.001
Type 2	2.15	117.707	<0.001

Significance tests for the non-parametric smooth terms from a generalized additive model of incident heart failure events on age, sex, deprivation, diabetes type and calendar year, with interaction terms included where these improved model fit, using a log-link and Poisson likelihood, with correction of the standard errors for overdispersion. Penalized thin plate regression splines were used to model non-linear associations for calendar year by diagnosis type. Predictions were made for men and women aged 50.

Table S11, Heart failure case-fatality model, coefficients and standard errors

	Estimate	Std. Error	z value	Pr(>|z|)
(Intercept)	-2.938	0.037	-79.507	<0.001
age_ten	0.239	0.024	10.07	<0.001
I(age_ten^2)	0.041	0.005	7.985	<0.001

	Estimate	Std. Error	z value	Pr(>|z|)
sex	0.041	0.02	2.077	0.038
dep_two	0.113	0.016	7.317	<0.001
typet1dm	0.649	0.104	6.248	<0.001
typet2dm	-0.046	0.031	-1.483	0.138
sex:typet1dm	-0.375	0.158	-2.377	0.017
sex:typet2dm	0.021	0.044	0.485	0.628

Logistic regression model with death as the outcome and admission or death as the denominator. Age_ten is the age in years divided by ten and dep_two is the deprivation score divided by two.

Table S12, Heart failure case-fatality over time model, coefficients and standard errors

	Estimate	Std. Error	z value	Pr(>|z|)
(Intercept)	-2.917	0.037	-79.47	<0.001
age_ten	0.23	0.024	9.696	<0.001
I(age_ten^2)	0.044	0.005	8.521	<0.001
sex	0.036	0.018	2.064	0.039
dep_two	0.108	0.016	6.988	<0.001
year	-0.033	0.003	-10.135	<0.001
typet1dm	0.47	0.079	5.976	<0.001
typet2dm	-0.017	0.023	-0.768	0.442
year:typet1dm	0.01	0.027	0.38	0.704
year:typet2dm	-0.006	0.008	-0.817	0.414

Logistic regression model with death as the outcome and admission or death as the denominator. Age_ten is the age in years divided by ten and dep_two is the deprivation score divided by two.

Additional analysis - IHD admissions excluded

Table S13 Heart failure admissions and deaths by age, sex, deprivation and diagnosis with IHD admissions excluded

Age	Sex	Deprivation	Diagnosis	Admissions	Persontime
20 to 49	men	1-5	No diabetes	901	5069826
20 to 49	men	1-5	Type1	29	41407
20 to 49	men	1-5	Type 2	78	49272
20 to 49	men	6-10	No diabetes	1692	5263834
20 to 49	men	6-10	Type1	50	44543
20 to 49	men	6-10	Type 2	138	76831
20 to 49	women	1-5	No diabetes	397	5249540
20 to 49	women	1-5	Type1	diff	30878
20 to 49	women	1-5	Type 2	diff	31920
20 to 49	women	6-10	No diabetes	822	5601684
20 to 49	women	6-10	Type1	35	32785
20 to 49	women	6-10	Type 2	77	58653

Age	Sex	Deprivation	Diagnosis	Admissions	Persontime
50 to 59	men	1-5	No diabetes	1587	1693233
50 to 59	men	1-5	Type1	28	12659
50 to 59	men	1-5	Type 2	310	99493
50 to 59	men	6-10	No diabetes	2141	1458332
50 to 59	men	6-10	Type1	56	11988
50 to 59	men	6-10	Type 2	546	124173
50 to 59	women	1-5	No diabetes	588	1794491
50 to 59	women	1-5	Type1	24	8981
50 to 59	women	1-5	Type 2	117	57015
50 to 59	women	6-10	No diabetes	1123	1569973
50 to 59	women	6-10	Type1	47	8417
50 to 59	women	6-10	Type 2	246	87663
60 to 69	men	1-5	No diabetes	2834	1263927
60 to 69	men	1-5	Type1	50	7243
60 to 69	men	1-5	Type 2	871	145751
60 to 69	men	6-10	No diabetes	3678	1124789
60 to 69	men	6-10	Type1	63	6891
60 to 69	men	6-10	Type 2	1424	164762
60 to 69	women	1-5	No diabetes	1489	1411856
60 to 69	women	1-5	Type1	30	6061
60 to 69	women	1-5	Type 2	364	92946
60 to 69	women	6-10	No diabetes	2409	1287403
60 to 69	women	6-10	Type1	52	5749
60 to 69	women	6-10	Type 2	862	133206
70 to 79	men	1-5	No diabetes	3982	741231
70 to 79	men	1-5	Type1	45	3528
70 to 79	men	1-5	Type 2	1559	122825
70 to 79	men	6-10	No diabetes	4860	704115
70 to 79	men	6-10	Type1	72	3448
70 to 79	men	6-10	Type 2	1962	136811
70 to 79	women	1-5	No diabetes	3334	943974
70 to 79	women	1-5	Type1	49	3833
70 to 79	women	1-5	Type 2	1042	104267
70 to 79	women	6-10	No diabetes	5120	987247
70 to 79	women	6-10	Type1	67	4025
70 to 79	women	6-10	Type 2	1833	143606
80 to 89	men	1-5	No diabetes	5219	340043
80 to 89	men	1-5	Type1	19	929
80 to 89	men	1-5	Type 2	1284	50231
80 to 89	men	6-10	No diabetes	5336	314982
80 to 89	men	6-10	Type1	26	856
80 to 89	men	6-10	Type 2	1284	47422
80 to 89	women	1-5	No diabetes	8582	642195
80 to 89	women	1-5	Type1	28	1271
80 to 89	women	1-5	Type 2	1412	65181
80 to 89	women	6-10	No diabetes	9563	654989
80 to 89	women	6-10	Type1	46	1353
80 to 89	women	6-10	Type 2	1739	75363

Numbers less than or equal to 5, or where the difference from Table S1 is less than or equal to 5 were suppressed to maintain confidentiality.

Figure S6 Modelled rate of heart failure by diabetes type, age and sex with IHD admissions excluded

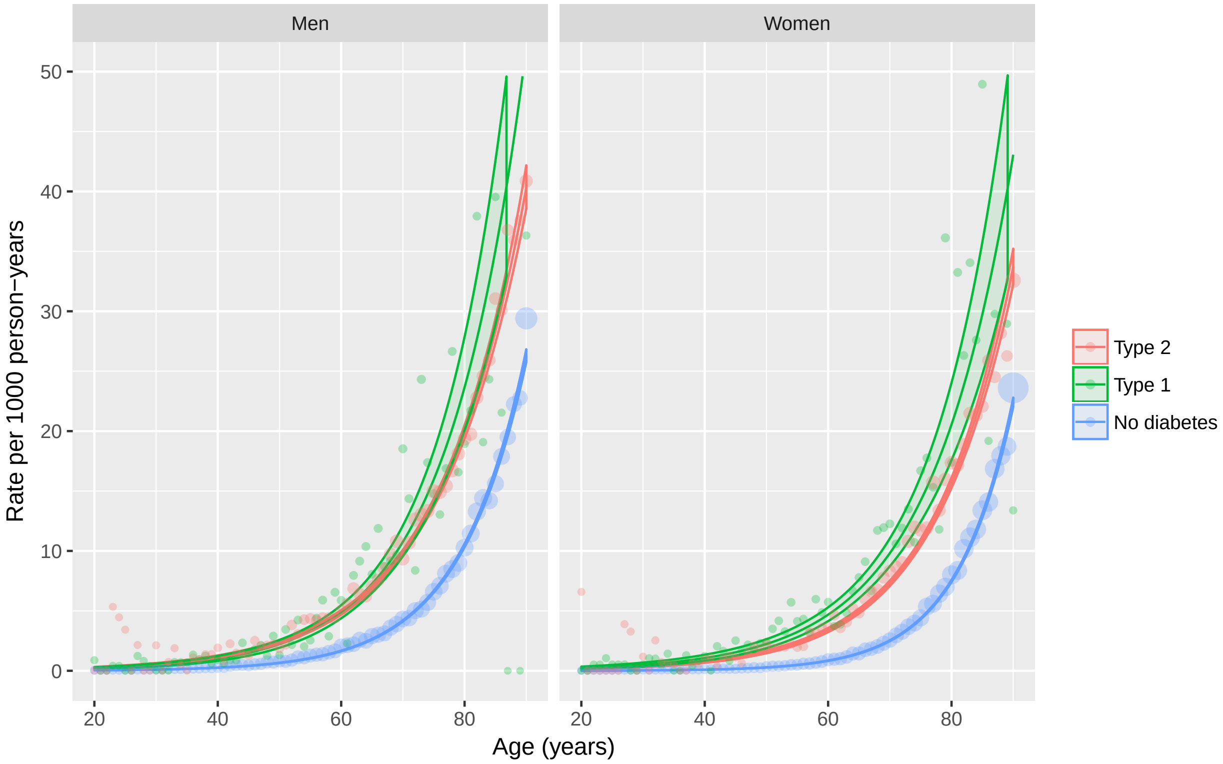

The lines represent the predicted rates obtained from quasi-Poisson regression models of incident heart failure events on age, sex, deprivation and diabetes type, with interaction terms included where these improved model fit. Predictions were made at the median deprivation score. Points represent event rates stratified by age (in years), sex and diabetes type.

Table S14 Cross-sectional model, coefficients and standard errors with IHD admissions excluded

	Estimate	Std. Error	t value	Pr(>|t|)
(Intercept)	-7.945	0.024	-326.852	<0.001
age_ten	1.079	0.009	124.583	<0.001
sex	-0.956	0.027	-36.019	<0.001
dep_two	0.621	0.017	36.802	<0.001
typet1dm	1.329	0.144	9.237	<0.001
typet2dm	1.481	0.062	23.86	<0.001
age_ten:sex	0.173	0.007	25.265	<0.001
age_ten:dep_two	-0.158	0.006	-26.279	<0.001
sex:dep_two	0.105	0.014	7.368	<0.001
age_ten:typet1dm	-0.222	0.068	-3.254	0.001
age_ten:typet2dm	-0.261	0.023	-11.256	<0.001
sex:typet1dm	0.855	0.108	7.927	<0.001
sex:typet2dm	0.43	0.049	8.763	<0.001
dep_two:typet1dm	-0.113	0.098	-1.161	0.246
dep_two:typet2dm	-0.187	0.043	-4.361	<0.001

	Estimate	Std. Error	t value	Pr(>|t|)
age_ten:sex:typet1dm	-0.221	0.052	-4.29	<0.001
age_ten:sex:typet2dm	-0.112	0.018	-6.158	<0.001
age_ten:dep_two:typet1dm	0.089	0.046	1.928	0.054
age_ten:dep_two:typet2dm	0.044	0.016	2.753	0.006

Quasi-Poisson regression model with admissions or death as the outcome. Age_ten is the age in years divided by ten and dep_two is the deprivation score divided by two. The standard errors and P-values are scaled to allow for overdispersion.

Figure S7 Trends in rates of heart failure by diabetes type, sex and calendar year with IHD admissions excluded

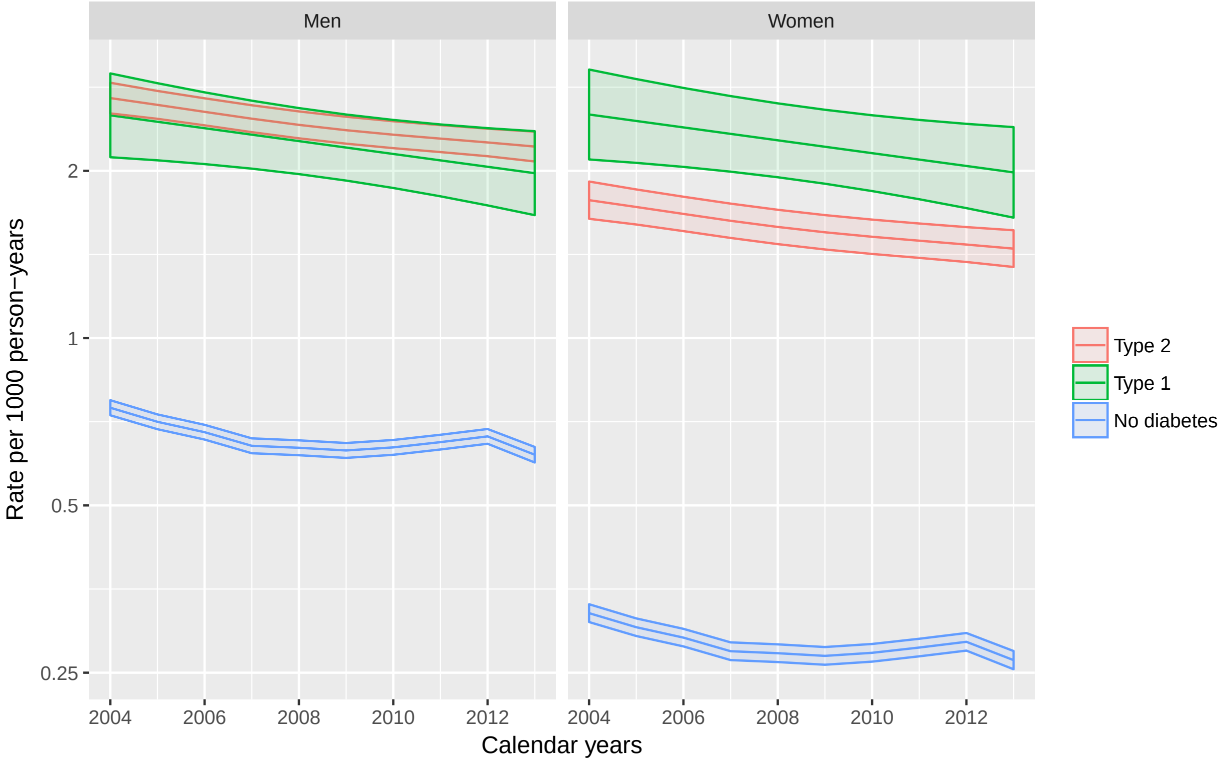

The lines represent the predicted rates obtained from generalized additive models of incident heart failure events on age, sex, deprivation, diabetes type and calendar year, with interaction terms included where these improved model fit, using a log-link and Poisson likelihood, with correction of the standard errors for overdispersion. Penalized thin plate regression splines were used to model non-linear associations for calendar year by diagnosis type. Predictions were made for men and women aged 50.

Table S15 Time trends model, coefficients and standard errors with IHD admissions excluded

	Estimate	Std. Error	t value	Pr(>|t|)
(Intercept)	-7.941	0.024	-327.026	<0.001

	Estimate	Std. Error	t value	Pr(>|t|)
age_ten	1.082	0.009	125.008	<0.001
sex	-0.957	0.026	-36.164	<0.001
dep_two	0.621	0.017	36.892	<0.001
year	-0.006	0.003	-2.127	0.033
typet1dm	1.343	0.144	9.36	<0.001
typet2dm	1.486	0.062	23.988	<0.001
age_ten:sex	0.172	0.007	25.252	<0.001
age_ten:dep_two	-0.158	0.006	-26.502	<0.001
sex:dep_two	0.105	0.014	7.426	<0.001
age_ten:year	-0.003	0.001	-3.302	0.001
age_ten:typet1dm	-0.224	0.068	-3.301	0.001
age_ten:typet2dm	-0.258	0.023	-11.183	<0.001
sex:typet1dm	0.854	0.107	7.955	<0.001
sex:typet2dm	0.428	0.049	8.763	<0.001
dep_two:typet1dm	-0.117	0.097	-1.197	0.231
dep_two:typet2dm	-0.187	0.043	-4.36	<0.001
year:typet1dm	-0.015	0.013	-1.15	0.25
year:typet2dm	-0.007	0.003	-2.2	0.028
age_ten:sex:typet1dm	-0.222	0.051	-4.335	<0.001
age_ten:sex:typet2dm	-0.112	0.018	-6.176	<0.001
age_ten:dep_two:typet1dm	0.089	0.046	1.932	0.053
age_ten:dep_two:typet2dm	0.045	0.016	2.79	0.005

Quasi-Poisson regression model with admissions or death as the outcome. Age_ten is the age in years divided by ten and dep_two is the deprivation score divided by two. The standard errors and P-values are scaled to allow for overdispersion.

Table S16 Estimates of non-parametric smooth functions with IHD admissions excluded

	Estimated degrees of freedom	Chi-squared	Approximate P-value
No diabetes	7.646	214.152	<0.001
Type 1	1.005	4.75	0.03
Type 2	1.968	66.125	<0.001

Significance tests for the non-parametric smooth terms from a generalized additive model of incident heart failure events on age, sex, deprivation, diabetes type and calendar year, with interaction terms included where these improved model fit, using a log-link and Poisson likelihood, with correction of the standard errors for overdispersion. Penalized thin plate regression splines were used to model non-linear associations for calendar year by diagnosis type.

Sensitivity analysis - Events coded in First position only

Figure S8 Modelled rate of heart failure by diabetes type, age and sex with diagnosis recored in first position only

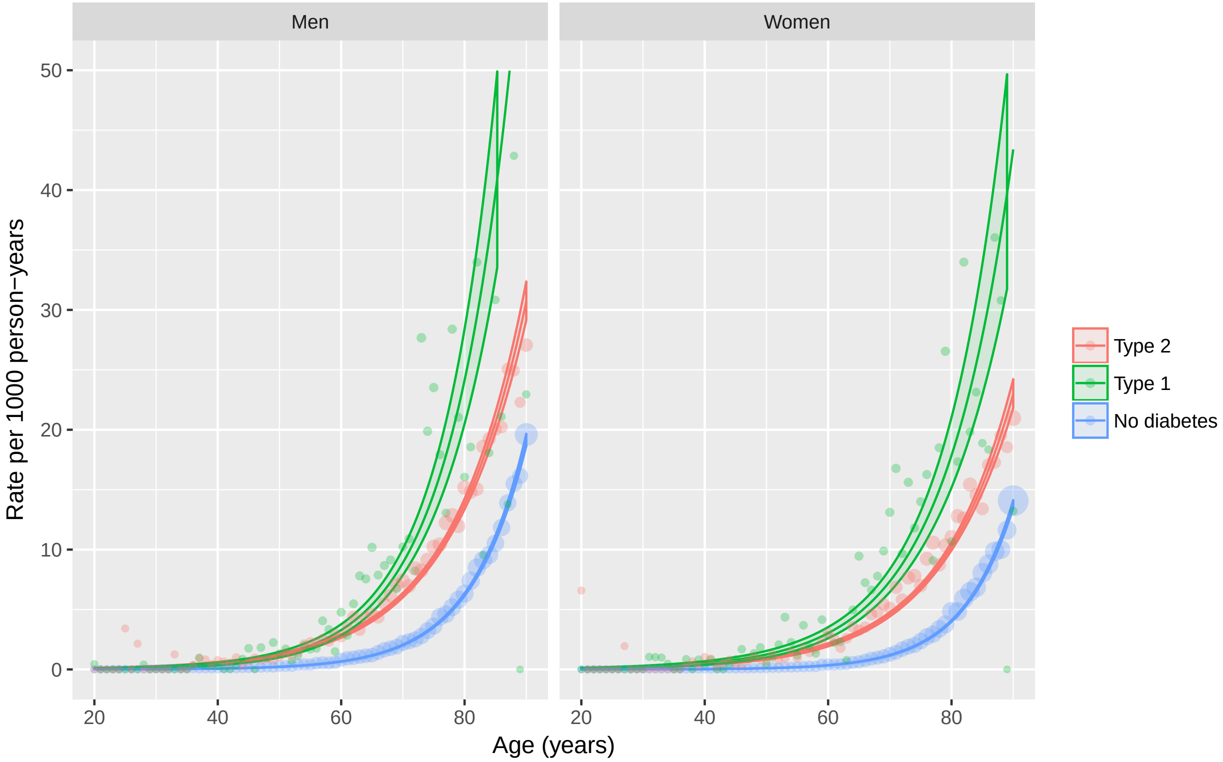

The lines represent the predicted rates obtained from quasi-Poisson regression models of incident heart failure events on age, sex, deprivation and diabetes type, with interaction terms included where these improved model fit. Predictions were made at the median deprivation score. Points represent event rates stratified by age (in years), sex and diabetes type.

Table S17 Cross-sectional model, coefficients and standard errors with diagnosis recored in first position only

	Estimate	Std. Error	t value	Pr(>|t|)
(Intercept)	-9.264	0.039	-236.959	<0.001
age_ten	1.319	0.013	100.519	<0.001
sex	-0.864	0.038	-22.468	<0.001
dep_two	0.828	0.026	31.31	<0.001
typet1dm	1.839	0.196	9.38	<0.001
typet2dm	2.031	0.082	24.651	<0.001
age_ten:sex	0.108	0.01	10.731	<0.001
age_ten:dep_two	-0.199	0.009	-22.317	<0.001
sex:dep_two	0.107	0.018	5.809	<0.001
age_ten:typet1dm	-0.173	0.083	-2.078	0.038
age_ten:typet2dm	-0.403	0.03	-13.568	<0.001
sex:typet1dm	0.785	0.14	5.613	<0.001
sex:typet2dm	0.446	0.063	7.124	<0.001

	Estimate	Std. Error	t value	Pr(>|t|)
dep_two:typet1dm	-0.119	0.13	-0.916	0.359
dep_two:typet2dm	-0.28	0.056	-4.996	<0.001
age_ten:sex:typet1dm	-0.217	0.061	-3.537	<0.001
age_ten:sex:typet2dm	-0.102	0.023	-4.51	<0.001
age_ten:dep_two:typet1dm	0.05	0.056	0.902	0.367
age_ten:dep_two:typet2dm	0.083	0.02	4.095	<0.001

Quasi-Poisson regression model with admissions or death as the outcome. Age_ten is the age in years divided by ten and dep_two is the deprivation score divided by two. The standard errors and P-values are scaled to allow for overdispersion.

Figure S9 Trends in rates of heart failure by diabetes type, sex and calendar year with with diagnosis recored in first position only

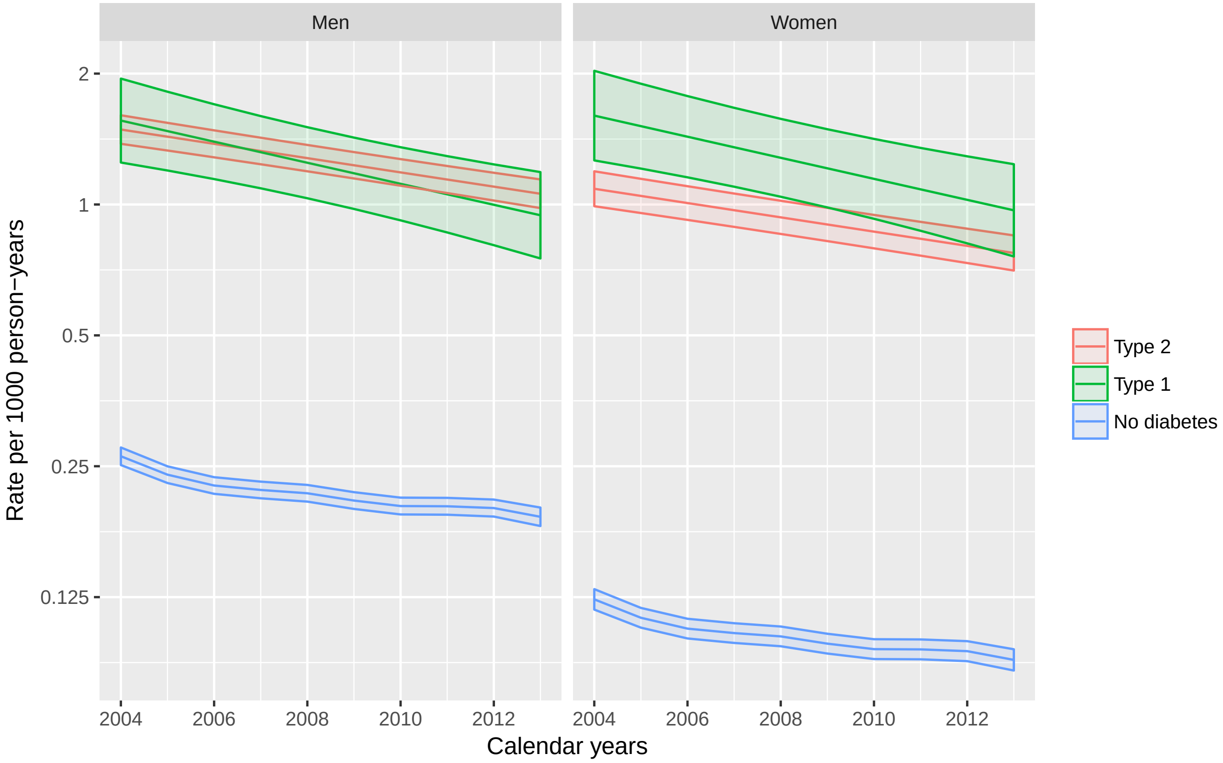

The lines represent the predicted rates obtained from generalized additive models of incident heart failure events on age, sex, deprivation, diabetes type and calendar year, with interaction terms included where these improved model fit, using a log-link and Poisson likelihood, with correction of the standard errors for overdispersion. Penalized thin plate regression splines were used to model non-linear associations for calendar year by diagnosis type. Predictions were made for men and women aged 50.

Table S18 Time trends model, coefficients and standard errors with diagnosis recored in first position only

	Estimate	Std. Error	t value	Pr(>|t|)
(Intercept)	-9.25	0.039	-237.045	<0.001
age_ten	1.322	0.013	100.86	<0.001
sex	-0.865	0.038	-22.57	<0.001
dep_two	0.826	0.026	31.325	<0.001
year	-0.03	0.004	-6.762	<0.001
typet1dm	1.863	0.195	9.548	<0.001
typet2dm	2.04	0.082	24.809	<0.001
age_ten:sex	0.107	0.01	10.652	<0.001
age_ten:dep_two	-0.2	0.009	-22.51	<0.001
sex:dep_two	0.108	0.018	5.877	<0.001
age_ten:year	0	0.002	-0.052	0.958
age_ten:typet1dm	-0.178	0.083	-2.152	0.031
age_ten:typet2dm	-0.401	0.03	-13.517	<0.001
sex:typet1dm	0.784	0.139	5.629	<0.001
sex:typet2dm	0.444	0.063	7.102	<0.001
dep_two:typet1dm	-0.125	0.129	-0.971	0.331
dep_two:typet2dm	-0.279	0.056	-4.974	<0.001
year:typet1dm	-0.025	0.015	-1.683	0.092
year:typet2dm	-0.007	0.004	-1.781	0.075
age_ten:sex:typet1dm	-0.219	0.061	-3.588	<0.001
age_ten:sex:typet2dm	-0.102	0.023	-4.512	<0.001
age_ten:dep_two:typet1dm	0.05	0.055	0.906	0.365
age_ten:dep_two:typet2dm	0.084	0.02	4.135	<0.001

Quasi-Poisson regression model with admissions or death as the outcome. Age_ten is the age in years divided by ten and dep_two is the deprivation score divided by two. The standard errors and P-values are scaled to allow for overdispersion.

Table S19 Estimates of non-parametric smooth functions with with diagnosis recored in first position only

	Estimated degrees of freedom	Chi-squared	Approximate P-value
No diabetes	5.772	294.872	<0.001
Type 1	1.01	15.662	<0.001
Type 2	1.018	127.99	<0.001

Significance tests for the non-parametric smooth terms from a generalized additive model of incident heart failure events on age, sex, deprivation, diabetes type and calendar year, with interaction terms included where these improved model fit, using a log-link and Poisson likelihood, with correction of the standard errors for overdispersion. Penalized thin plate regression splines were used to model non-linear associations for calendar year by diagnosis type.

Legend for interactive figure

The interactive figure is available at https://ihwph-hehta.shinyapps.io/dm_hf_fig2/.

This figure is an interactive version of Figure 2 which can be found in the main manuscript. The lines represent the predicted rates obtained from generalized additive models of incident heart failure events. The

ribbons are 95% confidence intervals. Covariates included in the model were age, sex, deprivation, diabetes type and calendar-year, with interaction terms included where these improved model fit. The model was fit with a log-link and Poisson likelihood, with correction of the standard errors for overdispersion. Penalized thin plate regression splines were used to model non-linear associations for calendar-year by diagnosis type.

References

1. Stef van Buuren, Karin Groothuis-Oudshoorn. MICE: Multivariate Imputation by Chained Equations in R. Journal of Statistical Software. 2011;45:1-67. DOI: 10.18637/jss.v045.i03.

2. Barnard, J. and Rubin, D.B. Small sample degrees of freedom with multiple imputation. Biometrika. 1999;86:948-955.
